# A novel long non-coding RNA connects obesity to impaired adipocyte function

**DOI:** 10.1016/j.molmet.2024.102040

**Published:** 2024-10-01

**Authors:** Aina Lluch, Jèssica Latorre, Núria Oliveras-Cañellas, Ana Fernández-Sánchez, José M. Moreno-Navarrete, Anna Castells-Nobau, Ferran Comas, Maria Buxò, José I. Rodríguez-Hermosa, María Ballester, Isabel Espadas, Alejandro Martín-Montalvo, Birong Zhang, You Zhou, Ralph Burkhardt, Marcus Höring, Gerhard Liebisch, Ainara Castellanos-Rubio, Izortze Santin, Asha Kar, Markku Laakso, Päivi Pajukanta, Vesa M. Olkkonen, José M. Fernández-Real, Francisco J. Ortega

**Affiliations:** 1 Institut d’Investigació Biomèdica de Girona (IDIBGI) – Girona, Spain; 2 CIBER de la Fisiología de la Obesidad y la Nutrición (CIBEROBN), Madrid, Spain; 3 School of Medicine, University of Girona (UdG), Girona, Spain; 4 Animal Breeding and Genetics Programme, Institute for Research and Technology in Food and Agriculture (IRTA), Torre Marimon, Caldes de Montbui, Spain; 5 Centro Andaluz de Biología Molecular y Medicina Regenerativa (CABIMER), Consejo Superior de Investigaciones Científicas (CSIC), University Pablo de Olavide, Seville, Spain; 6 CIBER de Diabetes y Enfermedades Metabólicas Asociadas (CIBERDEM), Madrid, Spain; 7 Systems Immunity Research Institute, Cardiff University, Cardiff, United Kingdom; 8 Institute of Clinical Chemistry and Laboratory Medicine, University Hospital Regensburg, Regensburg, Germany; 9 Universidad del País Vasco/Euskal Herriko Unibertsitatea (UPV/EHU), Bizkaia, Spain; 10 Ikerbasque, Basque Foundation for Science, Bilbao, Spain; 11 Instituto de Investigación Sanitaria Biocruces Bizkaia, Bizkaia, Spain; 12 Bioinformatics Interdepartmental Program, UCLA, Los Angeles (CA), USA; 13 Department of Human Genetics, David Geffen School of Medicine at UCLA, Los Angeles (CA), USA; 14 Department of Medicine, University of Eastern Finland and Kuopio University Hospital, Kuopio, Finland; 15 Institute for Precision Health, David Geffen School of Medicine at UCLA, Los Angeles (CA), USA; 16 Minerva Foundation Institute for Medical Research, University of Helsinki, Helsinki, Finland

**Keywords:** linc-GALNTL6-4, Adipose tissue, Adipocytes, Triglycerides, Obesity

## Abstract

**Background:**

Long non-coding RNAs (lncRNAs) can perform tasks of key relevance in fat cells, contributing, when defective, to the burden of obesity and its sequelae. Here, scrutiny of adipose tissue transcriptomes before and after bariatric surgery (GSE53378) granted identification of 496 lncRNAs linked to the obese phenotype. Only expression of linc-GALNTL6-4 displayed an average recovery over 2-fold and FDR-adjusted p-value <0.0001 after weight loss. The aim of the present study was to investigate the impact on adipocyte function and potential clinical value of impaired adipose linc-GALNTL6-4 in obese subjects.

**Methods:**

We employed transcriptomic analysis of public dataset GSE199063, and cross validations in two large transversal cohorts to report evidence of a previously unknown association of adipose linc-GALNTL6-4 with obesity. We then performed functional analyses in human adipocyte cultures, genome-wide transcriptomics, and untargeted lipidomics in cell models of loss and gain of function to explore the molecular implications of its associations with obesity and weight loss.

**Results:**

The expression of linc-GALNTL6-4 in human adipose tissue is adipocyte-specific and co-segregates with obesity, being normalized upon weight loss. This co-segregation is demonstrated in two longitudinal weight loss studies and two cross-sectional samples. While compromised expression of linc-GALNTL6-4 in obese subjects is primarily due to the inflammatory component in the context of obesity, adipogenesis requires the transcriptional upregulation of linc-GALNTL6-4, the expression of which reaches an apex in terminally differentiated adipocytes. Functionally, we demonstrated that the knockdown of linc-GALNTL6-4 impairs adipogenesis, induces alterations in the lipidome, and leads to the downregulation of genes related to cell cycle, while propelling in adipocytes inflammation, impaired fatty acid metabolism, and altered gene expression patterns, including that of apolipoprotein C1 (APOC1). Conversely, the genetic gain of linc-GALNTL6-4 ameliorated differentiation and adipocyte phenotype, putatively by constraining APOC1, also contributing to the metabolism of triglycerides in adipose.

**Conclusions:**

Current data unveil the unforeseen connection of adipocyte-specific linc-GALNTL6-4 as a modulator of lipid homeostasis challenged by excessive body weight and meta-inflammation.

## Highlights

• The non-conserved expression of linc-GALNTL6-4 in adipose tissue is adipocyte-specific and opposite to obesity.
• Adipogenesis requires the transcriptional regulation of linc-GALNTL6-4, which reaches an apex in differentiated fat cells.
• Impaired adipocyte-specific linc-GALNTL6-4 levels are mainly due to the inflammatory state in the context of obesity.
• The loss of linc-GALNTL6-4 in adipocytes displays altered lipidomes and the downregulation of genes related to cell cycle*.*
• While propelling inflammation, defective linc-GALNTL6-4 may impair FA metabolism and the expression of APOC1.
• In engineered adipocytes, increased linc-GALNTL6-4 compels changes related to FA sensing and an improved lipid landscape.

## Graphical abstract

**Figure:**
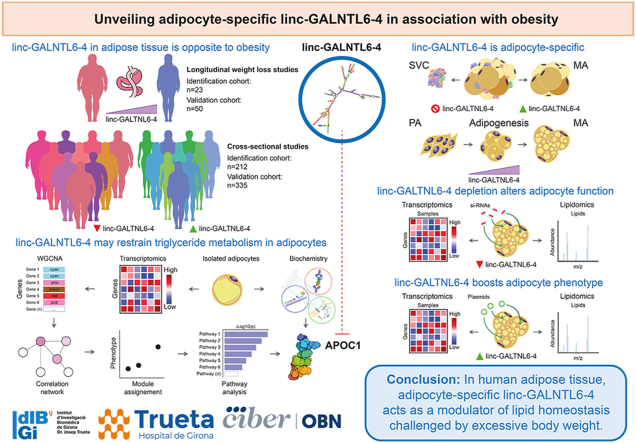

## 1 Introduction

During the last few decades, second-generation sequencing has revealed that the main body of the human genome is transcribed into molecules of ribonucleic acid (RNA) that do not code for proteins [1,2]. Among these, ∼27% are long non-coding (lnc)RNAs with sequences scarcely conserved during evolution [3] and mostly expressed in a tissue-specific manner [[4], [5], [6]]. Accounting for over 68% of bulk human transcriptomes [7], the widespread activity of lncRNAs hinges on their interaction with DNA, RNA and/or proteins [8]. Through these interactions, lncRNAs have emerged as scaffold components of nuclear architecture [9], regulators of chromosome conformation and transcription factor recruitment [10], and modifiers of messenger (m)RNA synthesis and stability, as well as its final translation into functional proteins [11,12]. Located within the nucleus, or contained in the cytoplasm of a large variety of mammalian cells, lncRNAs perform a broad range of functions in differentiation and development [13], also contributing to the burden of disease [14,15]. On this subject, the most recent research has shown how important the epigenetic regulation exercised by lncRNAs can be for adipocyte biology and function [16], also participating in the pathophysiology of obesity [17,18]. As a result, it is now recognized that deranged lncRNA expression patterns in adipose tissue may engage a range of metabolic disturbances associated with an obese phenotype [19]. However, identification and functional evaluation of specific adipose-derived lncRNAs related to obesity and its clinical sequelae are still far from complete. The current study stems from the analysis of bulk transcriptomes of human adipose tissue before and after weight loss, which allowed the identification of linc-GALNTL6-4, a novel adipocyte-specific lncRNA only found in humans and showing expression levels steeply increased upon the loss of fat. Scrutiny of additional fat samples further confirmed that this unique lncRNA opposes body weight, is solely present in the adipocyte cell fraction of adipose tissue, and is increased during the course of adipocyte differentiation. Observations made in human patient cohorts and functional experiments carried out on cultured adipocytes suggest that linc-GALNTL6-4 regulates the commitment of adipocytes towards the control of APOC1 levels and appropriate lipid patterns, potentially entangling adipose tissue function in metabolic homeostasis and dyslipidaemia.

## 2 Results

### 2.1 Expression of linc-GALNTL6-4 in adipose tissue is inverse to obesity

**Figure 1:**
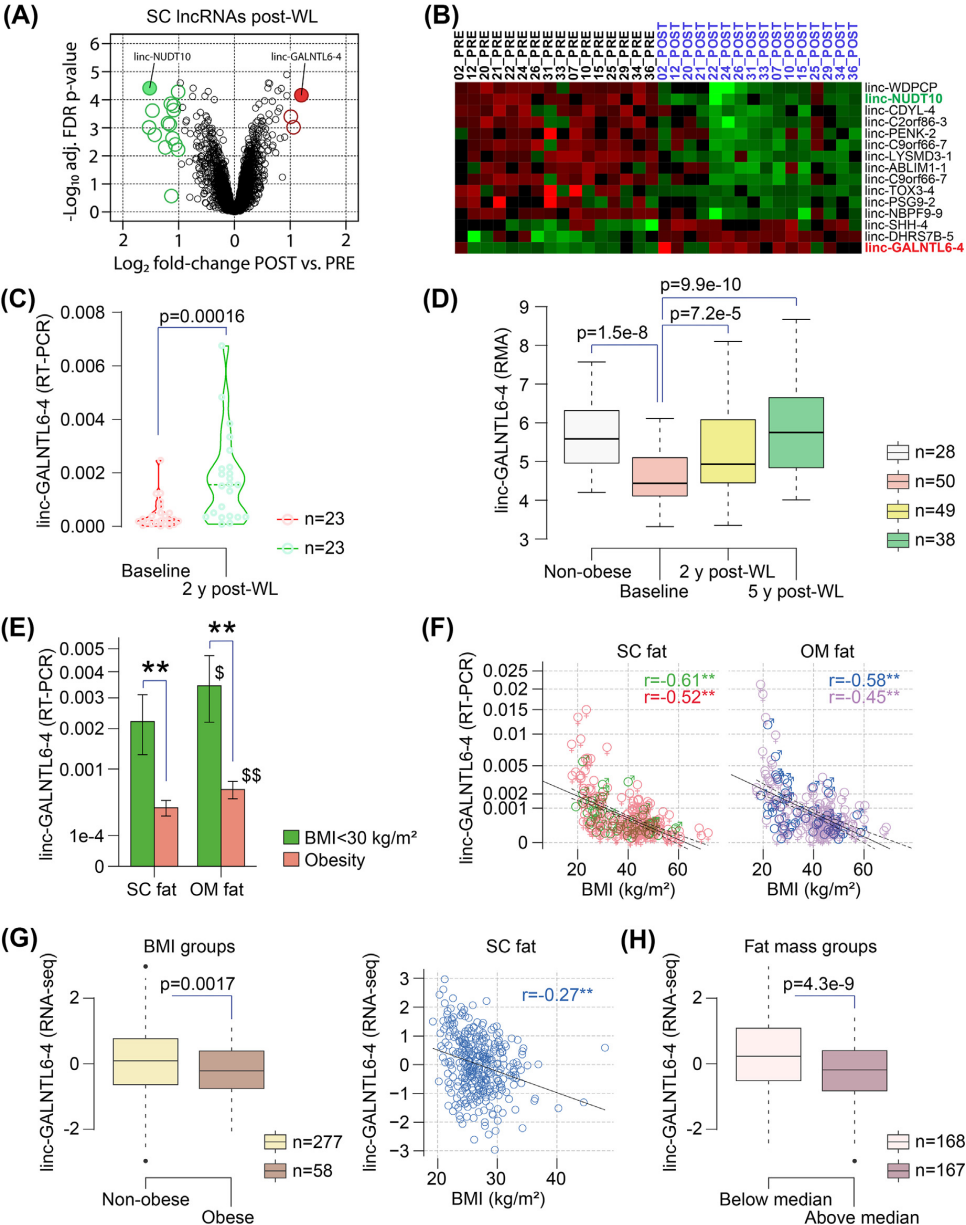
linc-GALNTL6-4 in adipose tissue is opposite to obesity. **(A)** Volcano plot of upregulated (red) and downregulated (green) lncRNAs in SC adipose tissue after weight loss (n = 16), as per inclusion criteria noted in **Methods**, and listed in **(B)**. Dynamic adaptations affecting adipose linc-GALNTL6-4 after weight loss were confirmed by means of **(C)** real time PCR (n = 23), and in **(D)** an independent dataset of 50 participants (microarray) [24]. linc-GALNTL6-4 levels are provided as box plots of 75th to 25th percentiles with the median, and whiskers at maximum and minimum values. **(E)** Mean and S.D. for linc-GALNTL6-4 values in SC (for obese and non-obese (BMI<30 kg/m^2^) subjects, n = 73 and 136, respectively) and OM (n = 59 and 111) fat samples from an independent cohort of 212 participants, and in **(F)** association with BMI. ∗∗p < 0.001 for comparisons between groups of subjects segregated according to their BMI, and ^$^p < 0.05 and ^$$^p < 0.001 for comparisons of OM vs SC. Coloured numbers depict Spearman's rank (r) correlations for men (green and blue) and women (red and purple), and ∗∗p < 0.001. Continuous and staggered black lines show regression and 95% confidence interval. Plots **(G)** and **(H)** depict linc-GALNTL6-4 values in the METSIM study, a population-based study including bulk RNA-seq in the SC fat of 335 Finnish men, in association with BMI and fat mass, respectively. (For interpretation of the references to color in this figure legend, the reader is referred to the Web version of this article.)

To identify transcripts correlating with adipose tissue function, expression profiling of 16 severe obese women undergoing bariatric surgery was performed. Anthropometric and biochemical phenotyping of these participants is shown in Table S1 and has been reported previously [20]. Microarray analysis depicted a total of 5,018 transcripts differentially expressed (DE) when comparing subcutaneous (SC) adipose tissue at the baseline and ∼2 years after surgery-induced weight loss (adjusted false discovery rate (FDR) p-value<0.05) [20]. Among these, 496 transcripts were defined as putative lncRNAs, after excluding pseudogenes as well as spurious and uncharacterized ncRNAs, based on manual annotation [21]. Two hundred and eighty-two (56.9%) of the lncRNAs with dynamic changes were upregulated, while expression of 214 candidates decreased upon the loss of weight (Figure 1A). Only two lncRNAs displayed an average fold-change > [2] and adjusted FDR p-value<0.0001: linc-GALNTL6-4 (also known as LINC01612, RP11-789C1.1, XLOC_003775, and TCONS_00008319), and linc-NUDT10 (lnc-BMP15-1:1, XLOC_007982, TCONS_00017171, CATG00000111229.1, and ASMER-2 [22]). Expression of linc-NUDT10 was diminished, whereas linc-GALNTL6-4 values in paired pre-post surgery samples displayed a notable increase (Figure 1B). While the decay of adipose-derived linc-NUDT10 in obese patients upon weight loss was documented in 2018 by Zhang and co-workers [23], we were struck by the unprecedented regulation of linc-GALNTL6-4, and hence we focused our research on this lncRNA. First, we confirmed the hitherto overlooked expression of linc-GALNTL6-4 in human adipose tissue. Results collected from the Genotype-Tissue Expression (GTEx) Analysis Release V8 (dbGaP Accession phs000424.v8.p2) showed consistent expression of linc-GALNTL6-4 in SC (n = 663) and visceral (great omentum; n = 541) adipose tissue, ranking up to 9 and 20 transcripts per million (TPM), respectively (Fig. S1a). Additionally, co-expression network analysis of linc-GALNTL6-4 and other RNAs of reference [20] pointed that inter-individual variations affecting this lncRNA were associated with concomitant fluctuations in 181 mRNAs (Table S2). Interpretation of the available annotations by Ingenuity Pathways Analysis (IPA) and g:Profiler highlighted the enrichment of genes related to energy handling, including fatty acid and carbohydrate metabolism (Table S3). Then, we did real time PCR assays in an extended sample of 23 obese patients following surgery-induced weight loss, including those of the discovery cohort (Table S1). In agreement with our microarray results, expression of SC linc-GALNTL6-4 rose (3.8-fold change) in fat samples obtained ∼2 years after gastric bypass (Figure 1C). To better substantiate the apparent regulation of adipose linc-GALNTL6-4, Robust Multichip Average (RMA) expression measures were retrieved from reference [24]. There, biopsies of SC adipose tissue were obtained by needle aspiration from 50 obese women at the baseline and 2 (n = 49) and 5 (n = 38) years after gastric bypass, as well as in a nonoperated group of 28 healthy weight women. Also in this independent dataset (Figure 1D), the utility of linc-GALNTL6-4 as a biomarker of dynamic adaptations affecting adipose tissue function was outlined by the increased expression found in age-matched non-obese subjects (1.24-fold) and obese patients following weight loss (1.16- and 1.28-fold change at 2 and 5 years after surgery, respectively). We queried next the abundance of linc-GALNTL6-4 in SC and omental (OM) fat samples of an independent cohort of individuals with and without obesity (body mass index (BMI) threshold of 30 kg/m^2^) (Table S4). In concordance with our longitudinal findings, cross-sectional comparisons confirmed the partial loss of SC and OM adipose linc-GALNTL6-4 in obese subjects (Figure 1E). Notably, linc-GALNTL6-4 levels, which were consistently higher in OM than in SC fat depots (Figure 1E), were inversely correlated with BMI (Figure 1F) and fasting glycaemia, and also opposite to the expression of leptin (*LEP*) and tumor necrosis factor alpha (*TNFα*), while running together with biomarkers of adipocyte function such as *FASN*, *ACACA*, *IRS1*, and *GLUT4* (Table S5). Additionally, multiple linear regression analyses highlighted that BMI greatly contributed to the variance of SC and OM linc-GALNTL6-4, after controlling for gender, age and fasting glucose (Table S5). For a replication of these cross-sectional observations, we employed RNA sequencing (RNA-seq) data from the subcutaneous fat of 335 male subjects (Table S6) in the Finnish METSIM cohort [[25], [26], [27]]. Here, the expression of linc-GALNTL6-4 showed a significant inverse trend with BMI (Figure 1G) and % fat mass (Figure 1H), lending further, independent validation to the detected association of impaired linc-GALNTL6-4 expression levels with obesity and biomarkers of adipocyte function (Table S7). On the basis of these lines of evidence, we hypothesized that, in human adipose tissue, the loss of linc-GALNTL6-4 might be relevant for energy handling and obesity-associated adipocyte dysfunction, and thus play a role in the set of metabolic disturbances that string along with obesity.

### 2.2 Expression of linc-GALNTL6-4 as a marker of adipocyte phenotype

**Figure 2:**
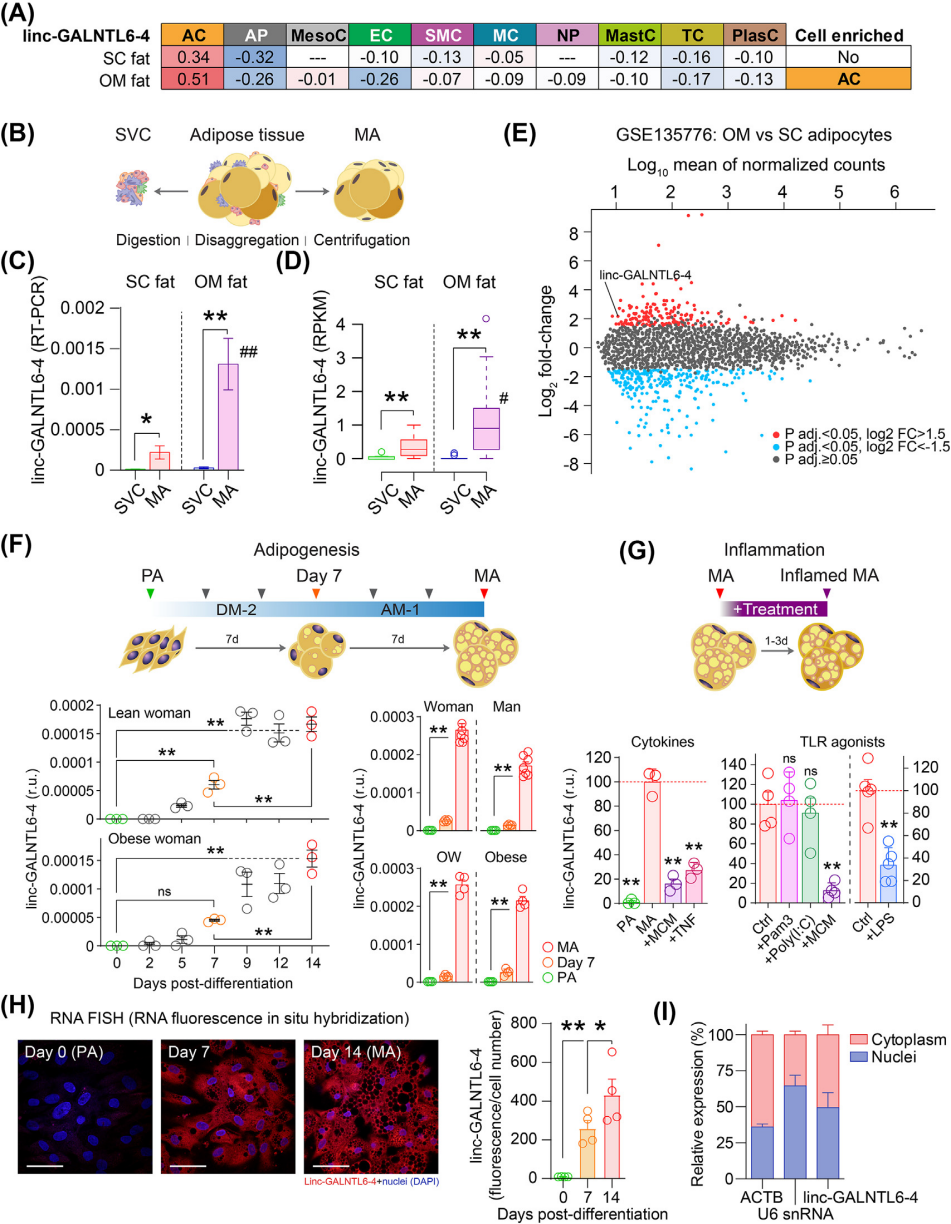
linc-GALNTL6-4 is adipocyte-specific. **(A)** Heatmap of pairwise Spearman correlation coefficients for linc-GALNTL6-4 (ENSG00000250266) and reference genes for different adipose tissue resident cell populations (see also in Fig. S1B). The lack of values for mesothelial cells (MesoC) and neutrophils (NP) is due to the low presence of these cell types in SC fat, as explained in reference [28]. **(B)** Digestion of adipose tissue and the study of disaggregated adipose-derived stromal vascular cells (SVC) and mature adipocytes (MA) further confirmed **(C)** the prevalent expression of linc-GALNTL6-4 in the latter. Single-fat cell RNA-seq measures (RPKM) of linc-GALNTL6-4 in the dataset with ascension number GSE135776 [30] also confirmed **(D)** its most prominent expression in MA and **(E)** the higher amounts observed in OM adipocytes, when compared to SC adipocytes. **(F)** Increased linc-GALNTL6-4 expression was observed in cultures of preadipocytes (PA) while differentiating into lipid-containing MA. DM-2 (first) and AM-1 (second week) stand for differentiation and adipocyte media, respectively (see in **Methods**). In an independent assay **(g)**, expression of linc-GALNTL6-4 was measured in differentiating adipocytes and in MA when challenged with a macrophage LPS-conditioned media (MCM). **(H)** RNA Fluorescence In Situ Hybridization (FISH) revealed increased cytoplasmic and nuclear linc-GALNTL6-4 in human PA while differentiating towards MA, **(I)** as further confirmed by real time PCR measures assessed in the nucleus and cytoplasm of MA. The scale bars denote 100 μm length in representative 20x immunofluorescent images. Plots show mean and S.E.M. Dots show results for each biological replicate (wells of the same 12-well plate). Statistical significance was assessed by ANOVA (post-hoc Bonferroni's multiple comparisons test) to assess the significance of dynamic changes in linc-GALNTL6-4 levels during adipogenesis, and two-tailed Student t-tests for comparisons treated versus control adipocytes. ns, not significant; ∗ and ^#^p < 0.05, ∗∗ and ^##^p < 0.001.

The above findings suggested that linc-GALNTL6-4 in adipose tissue is related to the biosynthetic nature of mature adipocytes (MA). As a matter of fact, the abundance of linc-GALNTL6-4 was primarily associated with the expression of adipocyte cell (AC)-specific genes (i.e., *ADIPOQ*, *LIPE*, and *PLIN1*) within depots of SC and OM adipose tissue (Figure 2A), independently of gender and location (Fig. S2b), and opposite to the adipocyte progenitor (AP) panel (*FKBP10*, *COL6A1*, and *COL6A2*) provided by Norreen-Thorsen et al. [28]. To better investigate this, expression of linc-GALNTL6-4 was evaluated in *ex vivo* isolated MA and the stromal vascular cell (SVC) fraction of SC and OM adipose tissue samples (Figure 2B). Notably, the abundance of this lncRNA occurred in the MA (Figure 2C), characterized by the buoyancy property of lipid-filled fat cells [29], while this transcript was nearly absent (Ct values ≥ 37) in SVC. In consonance with this, the single-cell RNA-seq measures of linc-GALNTL6-4 obtained by Vijay and co-workers [30] were also suggestive of the appearance of this lncRNA in MA (Figure 2D), and confirmed the higher expression observed in fat cells isolated from the great omentum (Figure 2E), when compared to SC adipocytes. Next, we examined in primary preadipocytes (PA) dynamic adaptations during *in vitro* differentiation. Accordingly, fat precursor cells from different donors revealed, regardless of sex and weight, steady increased amounts of linc-GALNTL6-4, with expression levels reaching an apex in terminally differentiated, lipid-filled adipocytes at day 14 after hormonal induction (Figure 2F and **S2a-b**). Also in agreement with our previous findings, the meta-analysis of publicly available transcriptomic studies targeting *in vitro* adipogenesis and the maintenance of an adipocyte phenotype [31,32] showed the highest amounts of linc-GALNTL6-4 in fully differentiated adipocytes (over 5 counts), matching the expression pattern of adipocyte markers and genes involved in lipid handling (Figures S2c-d). Then, we challenged cultures of MA with macrophage lipopolysaccharide (LPS)-conditioned medium (MCM), containing many type 1 macrophage-derived cytokines that can orchestrate metabolic inflammation [33], recombinant TNFα, LPS (TLR4 agonist), and other agonists for toll-like rectors (TLR), such as Pam3CSK4 (TLR1/2) and Poly(I:C) (TLR3) [34,35]. Treatments of MCM, as well as TNF and LPS alone (but not TLR1/2 or TLR3 agonists), dampened the expression of linc-GALNTL6-4 in adipocytes (Figure 2G), suggesting that its reduction in obese subjects is mainly driven by inflammatory changes affecting the adipose tissue, primarily characterized by increased extracellular lipid concentrations, deranged TNF signalling, adipocyte and macrophage induction by TLR4 through nuclear factor-kappaB (NF-kappaB) activation, and local macrophage accumulation [36]. Finally, we wanted to know the intracellular location of this lncRNA in human adipocytes. To this end, we performed RNA fluorescent *in situ* hybridization (FISH), which showed staining for linc-GALNTL6-4 in both the nucleus and the cytoplasm of MA (Figure 2H), also confirmed by real time PCR (Figure 2I). This approach further validated increased linc-GALNTL6-4 during the course of adipocyte differentiation (Figure 2H). Altogether, our compiled clinical and cellular data provided a body of *in vivo* and *in vitro* observations that bound altered expression of adipocyte-specific linc-GALNTL6-4 to the burden of obesity, inflammation, and ensuing fat cell dysfunction.

### 2.3 Defective linc-GALNTL6-4 impairs adipogenesis and alters adipocyte phenotype

**Figure 3:**
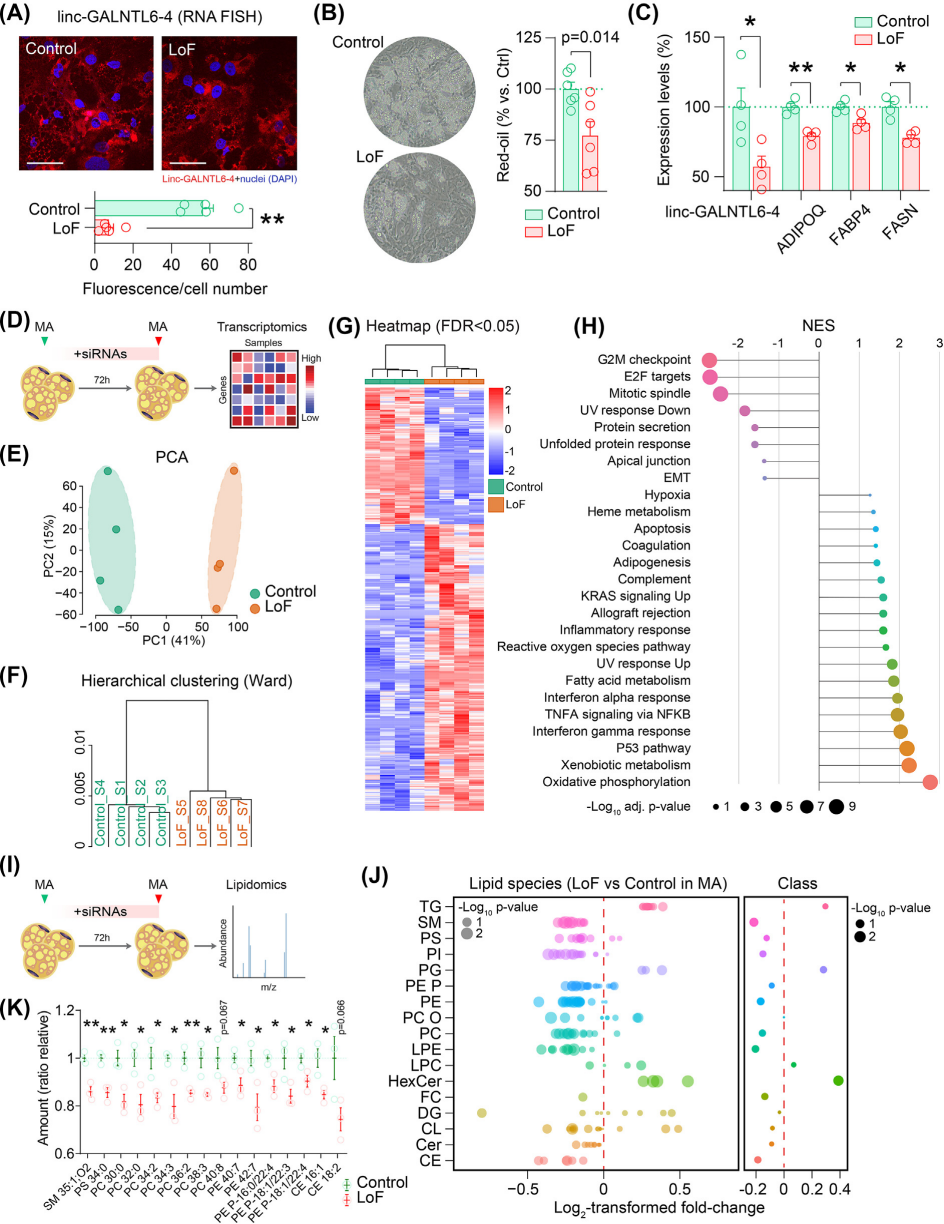
linc-GALNTL6-4 depletion alters adipocyte function. **(A)** Decreased linc-GALNTL6-4 signal (FISH) in adipocytes challenged with sh-RNA lentiviral particles drove impaired adipogenesis, as depicted by decreased **(B)** lipid droplets content and **(C)** expression of adipogenic markers (mean and S.E.M.; ∗p < 0.05 and ∗∗p < 0.001). The scale bars denote 100 μm length in representative 20x immunofluorescent images. **(D)** Schematic representation of our experimental approach to evaluate the loss of linc-GALNTL6-4 (LoF) in MA. **(E)** Principal component analysis (PCA), **(F)** hierarchical clustering, and **(G)** the heatmap of DE genes highlighted a great consistency within groups. **(H)** Gene Set Enrichment Analysis (GSEA) of Human Molecular Signatures Database (MSigDB) categories altered in our genetic LoF model. In an independent experiment **(I)**, the knockdown of linc-GALNTL6-4 modified the **(J)** lipid landscape of MA, with major effects on a number of **(K)** SM, PS and PC species. Plots show mean and S.E.M. Dots show results for each biological replicate (wells of the same 12-well plate). Statistical significance was assessed by two-tailed Student t-test. ∗p < 0.05, ∗∗p < 0.001.

To investigate the functional relevance of linc-GALNTL6-4 in adipocytes, we first employed a strategy of genetic loss-of-function (LoF). To this end, we infected cultures of primary human PA with lentiviruses expressing non-targeting short-hairpin RNAs (sh-RNAs), or sh-RNAs targeting linc-GALNTL6-4. After enrichment of transduced cells (puromycin selection), adipocyte precursors expressing sh-RNAs were induced to differentiate. This approach led to cultures of differentiated adipocytes with reduced expression of linc-GALNTL6-4 in the cytoplasm (Figure 3A). These cells displayed altered adipogenesis, as depicted by reduced accumulation of lipid droplets (Figure 3B) and decreased expression of genes related to the adipocyte phenotype (e.g., *ADIPOQ*, *FABP4*, and *FASN*) (Figure 3C). To decouple the potential relevance of linc-GALNTL6-4 to adipocyte biology from the partial blockage of adipogenesis, we conducted next LoF assays in already differentiated fat cells. To this end, cultures of MA were transfected with synthetic small interfering (si-)RNAs directed against linc-GALNTL6-4 (Figure 3D). In order to investigate the landscape of genes regulated by this treatment, we performed deep RNA-seq on cells challenged with si-linc-GALNTL6-4 (LoF) and non-targeting controls. Principal component analysis (Figure 3E) and hierarchical clustering (Figure 3F) showed a high degree of variation, separating the adipocytes challenged with oligonucleotides against linc-GALNTL6-4 and the control cells into distinct clusters. Gene set enrichment analysis (GSEA) [37] of messenger RNAs with most remarkable different expression (fold-change > [1.2] and adjusted FDR p-value<0.05) (Figure 3G) provided evidence of major changes, affecting pathways ranging from impaired cell-cycle control (e.g., G2M checkpoint, Mitotic spindle, and E2F targets) to enhanced oxidative phosphorylation, fatty acid metabolism, and inflammatory response (Figure 3H). To better endorse the impact of defective linc-GALNTL6-4 in adipocytes, we applied lipidomics to investigate the remodelling of adipocyte lipids in response to si-RNAs directed against linc-GALNTL6-4 (Figure 3I). This analysis showed a lipid pattern in LoF adipocytes quite distinct from control cells, mainly characterized by compositional changes in long-chain triglycerides (e.g. increased TG 55:2, 55:5, 56:6, 56:7, 60:2, and 60:3), the enrichment of hexosylceramides (HexCer), and the underrepresentation of sphingomyelins (SM), phosphatidylserines (PS), phosphatidylcholines (PC), and phosphatidylethanolamines (PE) (Figure 3J–K), further underscoring the effects of linc-GALNTL6-4 knockdown on adipocyte phenotype and lipid metabolism. Of note, examination of the adipocyte lipidome upon inflammatory conditions (the presence of macrophage-derived cytokines, dampened linc-GALNTL6-4 expression) indicated a shift in lipid composition mostly characterized by increased TG and HexCer, and a reduction of SM, PS and PE (Figures S3a-c, S4a-b). To summarize, the body of transcriptomic changes and alterations in the lipidomic landscape of MA with linc-GALNTL6-4 knocked-down indicated that variant expressions of this lncRNA in fat cells, and thus in bulk adipose tissue, may impair adipogenic commitment and alter the fat cell phenotype and lipid handling.

### 2.4 Increased linc-GALNTL6-4 shows positive effects in adipocytes

**Figure 4:**
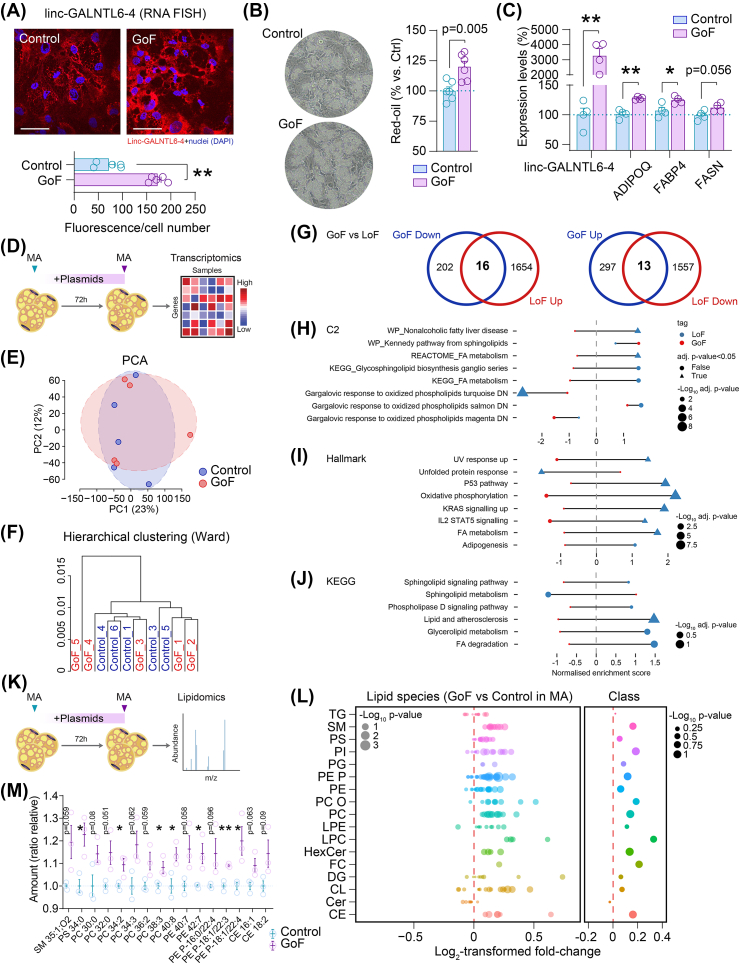
Increased linc-GALNTL6-4 boosts adipocyte phenotype. **(A)** Enhanced linc-GALNTL6-4 signal (FISH) resumes improved adipogenesis, as shown by **(B)** the amount of lipid droplets and **(C)** expression of adipogenic markers in differentiated human PA (mean and S.E.M.; ∗p < 0.05, ∗∗p < 0.001). The scale bars denote 100 μm length in representative 20x immunofluorescent images. **(D)** Schematic representation of the protocol used to evaluate the impact of linc-GALNTL6-4 (GoF) in human MA. **(E)** PCA and **(F)** hierarchical clustering indicated poor sample aggregation in treatment and control, but a hub of 29 transcripts depicted opposite changes in our models of GoF and LoF, as pointed in **(G)**. Forest plots show overall suppression or activation of signalling pathways in response to linc-GALNTL6-4 knockdown (LoF) and overexpression (GoF), as disclosed by integrative analysis of our transcriptomic data using **(H)** C2 and **(I)** Hallmark gene set collections of the Molecular Signature Database (MSigDB), and **(J)** the Kyoto Encyclopaedia of Genes and Genomes (KEGG). LoF vs control is inked in blue, and red indicates comparisons in GoF vs control. Circles and triangles show non-significant (adj. p-value>0.05) and significant results, respectively. The size of each symbol stands for the -Log_10_ adj. p-value. Also using quantitative lipidomics **(K)**, an opposite effect in the GoF model was revealed with regard to the LoF **(L)**, including significant impacts on a number of key lipid species, as shown in **(M)**. Plots depict mean and S.E.M. Dots show results for each biological replicate (wells of the same 12-well plate). Statistical significance was assessed by two-tailed Student t-test. ∗p < 0.05, ∗∗p < 0.001. (For interpretation of the references to color in this figure legend, the reader is referred to the Web version of this article.)

In parallel to our LoF, PA from same donor and fat depot (abdominal SC adipose tissue) were transfected with a plasmid coding for linc-GALNTL6-4 or a control mock vector during differentiation. In this model of genetic gain-of-function (GoF) (Figure 4A), overexpression of linc-GALNTL6-4 compelled opposite effects in the process of adipogenesis, with regard to the LoF: higher amounts of lipid droplets (Figure 4B) and slightly increased expression of adipocytic marker mRNAs than control (Figure 4C). Hence, the synthetic upregulation of this lncRNA appears to have a positive impact on the differentiation course of adipocyte progenitors into lipid-filled MA. However, when fully developed white adipocytes were transfected with these plasmids and prepared after three days for gene expression analysis (Figure 4D), neither PCA nor hierarchical clustering showed separation of the linc-GALNTL6-4 overexpressing and control cells (Figure 4E–F), indicating few changes in the bulk transcriptome. Following identification of specific coding transcripts modified upon linc-GALNTL6-4 GoF in MA (threshold set at fold-change > [1.2], and p-value<0.05), we focused on overlapping genes showing DE when compared to the LoF model (fold-change > [1.2], and adj. p-value<0.05). Amongst the coincidences observed, a 16-gene signature (*APOC1*, *ANKRD35*, *ARHGEF28*, *BBOX1, CHI3L1, COL21A1, EHD3, FOXQ1, HSPBAP1, IL33, INAFM1, MGST2, PAX4, RTN4RL2, SEMA3D,* and *SCN5A*) was characterized by an inverse expression pattern (i.e., down in GoF, up in LoF), while as many as 13 genes (*NASP, KLHL23, MAD2L1, BMP6, MYOZ2, POLR3A, B3GALNT1, DDIAS, SPATA5, LRRIQ1, ORC6, MT1A,* and *AUTS2*) were up-regulated upon linc-GALNTL6-4 GoF and down-regulated in the LoF model (Figure 4G). At the functional level, integrated pathway analysis of the GoF and LoF transcriptomic data was suggestive of opposing effects on fatty acid metabolism and sphingolipid signalling, which were under- or overrepresented in our antagonistic cell models (Figure 4H–J). Further supporting these observations, the overall abundance of many lipid species was slightly yet consistently increased in the GoF model, either when analysed in MA (Figure 4K–M and S4c) or in PA (Figure S3d-f and S4d). Such alterations included higher amounts of PS 34:2 in both PA and MA when subjected to linc-GALNTL6-4 GoF; PC 34:2, 38:3 and 40:8, and PE 42:7, P-18:1/22:3 and P-18:1/22:4 in MA (Figure 4M); and PC 30:0, PE P-16:0/22:3 and P-18:1/22:3, as well as lysophosphatidylethanolamines (e.g., LPE 20:3 and LPE 22:5), also modified in GoF PA systems (Fig. S3f). These changes may indicate that, additionally to strong alterations related to phenotype severity in adipocytes with reduced linc-GALNTL6-4, enhanced expression of this unique lncRNA may render stored fatty acids accessible for lipid remodelling, thus improving adipocyte commitment.

### 2.5 Adipose linc-GALNTL6-4-APOC1 axis may constrain triglyceridemia

**Figure 5:**
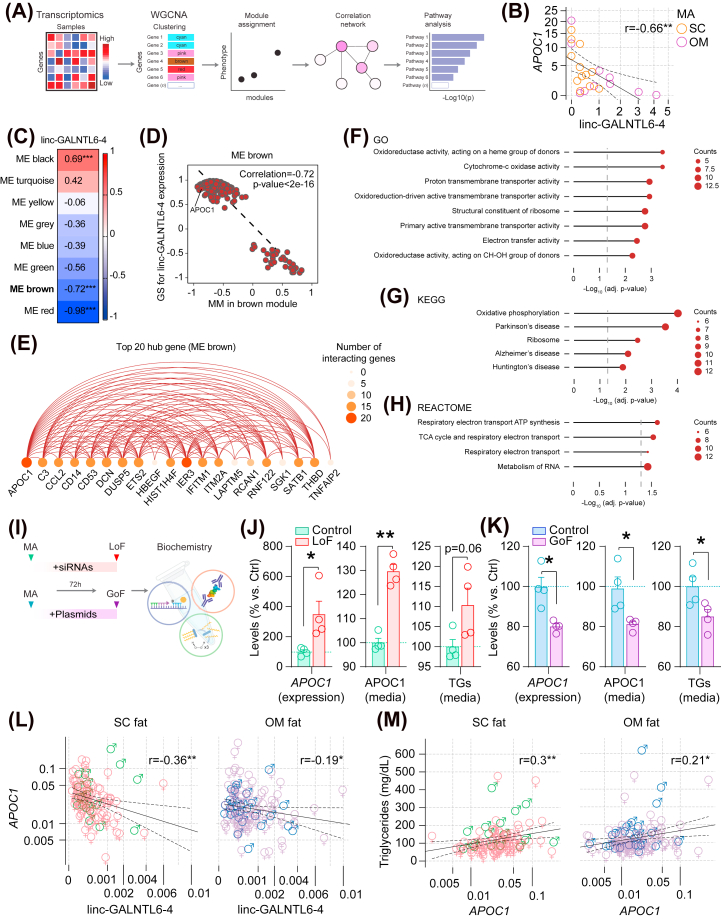
linc-GALNTL6-4 may restrain triglyceride metabolism in adipocytes. **(A)** General pipeline for Weighted Gene Correlation Network Analysis (WGCNA). **(B)** Linear regression analysis between RNA-seq APOC1 and linc-GALNTL6-4 RPKM values in SC and OM adipocytes [30]. **(C)** Heatmap of correlations between modules and linc-GALNTL6-4 expression levels (0 vs. >1 RPKM). Degrees of associations are indicted by colours, positive correlation, red; negative correlation, blue; no correlation, white. ∗∗∗p < 0.001. Example of this correlation is provided for the “brown” module in **(D)**, which included APOC1 and a hub of genes connected to this effector and linc-GALNTL6-4, as shown in **(E)**. Correlation coefficients and p-values between Module Membership (MM) and Gene Significance (GS) for all genes within each module were calculated using a linear regression model. The x-axis in **(D)** denotes the correlation between genes and modules, and the y-axis indicates the correlation between genes and linc-GALNTL6-4 expression levels. The size and colour intensity of each gene in **(E)** represent the number of interactions within the node. Forest plots show the hallmarks and pathways mostly characterized in human adipocytes for highly positive correlated genes with our selected lncRNA within the “brown” module, as interpreted by using bioinformatics resource of **(F)** GO, **(G)** KEGG, and **(H)** REACTOME. **(I)** Additional evaluation of our genetic models of LoF and GoF further validated the impact on APOC1 gene expression and protein levels, as measured by enzyme-linked immunoassays applied to the media, mirroring to some extent the triglycerides (TG) released by adipocytes when linc-GALNTL6-4 was **(J)** knocked-down (LoF) or **(K)** overexpressed (GoF). Data is presented as mean ± S.E.M. (n > 4 biological replicates/group); ∗p < 0.05, ∗∗p < 0.001. Scatter dot plots show **(L)** SC and OM APOC1 gene expressions opposite to linc-GALNTL6-4, and **(M)** directly associated with circulating TG, independently of sex, age and weight, as indicated by Spearman's rank (r)-order correlation tests. ∗p < 0.05, ∗∗p < 0.001. (For interpretation of the references to color in this figure legend, the reader is referred to the Web version of this article.)

We next performed weighted correlation network analysis (WGCNA) [38] to find clusters (modules) of genes associated with linc-GALNTL6-4 in isolated adipocytes (Figure 5A). These correlations facilitated a network-based gene screening method to validate previous associations and biological functions ascribed to the presence of this lncRNA in fat cells [30]. Intriguingly, of the list obtained when comparing DE genes affected in opposite directions in our GoF and LoF models, only APOC1 (Spearman's rho correlation coefficient r = −0.658, p-value = 0.00087) (Figure 5B), *INAFM1* (r = −0.486, p = 0.022), and BMP6 (r = −0.43, p = 0.046) displayed significant associations with linc-GALNTL6-4. To refine the gene clusters related to linc-GALNTL6-4, the overlap of DE genes and WGCNA modules was evaluated. This identified eight co-expression clusters (designated in Figure 5C as ‘ME colour’) in the dataset of fat cells showing>1 (n = 5) or 0 (n = 4, after discarding one outlier) linc-GALNTL6-4 mapped Reads Per Kilobase per Million (RPKM). Three of these modules showed significant association with linc-GALNTL6-4 (Figure 5C), and one of them (‘ME brown’) included *APOC1* (Figure 5D), which was highly connected to many other genes within this cluster (Figure 5E). Subsequently, GSEA was performed to evaluate whether genes in the ‘ME brown’ cluster are aligned with pathways of relevance in MA. Interestingly, they displayed significant (adjusted p-value<0.05) gene enrichment in specific hallmarks (Figure 5F–H), which were mostly indicative of variations affecting cellular respiration in adipocytes with high or low linc-GALNTL6-4 levels (Table S8). We next conducted additional LoF and GoF experiments, in which we studied the production of APOC1 and triglycerides by adipocytes with reduced or enhanced expression of linc-GALNTL6-4 (Figure 5I). We first confirmed by mRNA quantification and analysis of secreted APOC1 with an ELISA that APOC1 was inversely associated with the LoF or GoF of adipocyte linc-GALNTL6-4 (Figure 5J–K). As the release of adipose tissue APOC1 converges with delayed plasma clearance of dietary triglyceride-rich lipoproteins [39], we next assessed in our cell cultures the regulation of triglyceride secretion into the media. Notably, changes in APOC1 were concomitant with significant variations in triglycerides, which were increased in the media of MA with linc-GALNTL6-4 knocked down, while reduced levels of both occurred in the GoF model (Figure 5J–K). Finally, we examined the possible physiological significance of these associations by querying the human cohorts 1 and 2. Expression of APOC1 mRNA ran opposite to linc-GALNTL6-4 in both the SC and the OM depots (Figure 5L), independently of age, gender, BMI and other confounders. In addition, the concentration of triglycerides in blood, also polar to the expression of linc-GALNTL6-4 in fat samples (Table S5), was concomitant with APOC1 expression in both SC (adjusted R^2^ = 0.14, F-value = 0.001; β = 0.24, p-value = 0.011) and OM (R^2^ = 0.1, F-value = 0.002; β = 0.26, p-value = 0.003) adipose tissue (Figure 5M), independently of weight, sex and age (Table S9). Altogether, these compiled lines of evidence suggest that impaired linc-GALNTL6-4 in obese adipose tissue modifies lipid handling in adipocytes, and thus modulates the contribution of adipose tissue to circulating triglycerides.

## 3 Discussion

In comparison with other research topics in biomedicine, the study of lncRNAs lacks clear translational impact because of the inability to overcome the cross-species conservation gap (many lncRNAs show low expression levels and poor conservation across species [5]), limited knowledge of their biological function, and scarce information gathered in human samples. In the present study, we show that the expression of linc-GALNTL6-4 in human adipose tissue is adipocyte-specific and co-segregates with obesity, being normalized after weight loss. This co-segregation is demonstrated in two longitudinal weight loss studies and two cross-sectional studies. Furthermore, we present cellular and molecular insights showing that linc-GALNTL6-4 is relevant for adipocyte commitment, and that impaired expression levels in adipose tissue are mainly due to obesity and meta-inflammation. Our compiled evidence outlines a role for linc-GALNTL6-4 in regulating adipocyte transcriptomes and lipid landscapes, also contributing to fat cell differentiation. Our data reveal that modulation of linc-GALNTL6-4 impacts the adipocyte phenotype, as shown by multiple assays carried out in genetic models of loss and gain-of-function. Thereby, we demonstrate that linc-GALNTL6-4 controls a conserved genomic program involved in the performance of white adipocytes towards the regulation of fatty acid uptake, oxidative phosphorylation, and triglyceride metabolism, as well as the expression of APOC1. Our results suggest that the function of linc-GALNTL6-4 as a modulator of fatty acid sensing and adipocyte metabolism may be challenged by proinflammatory signals related to a large amount of body fat, further contributing to lipid abnormalities in patients suffering from obesity.

Alterations in lncRNA signatures have proved to be of importance in the context of linking the epigenetics of adipose tissue to energy homeostasis. An increasing number of original studies report the apparent role of lncRNAs in regulating fat cell commitment [16,19,40,41], affecting metabolism [42,43] and adipose tissue function [44,45]. Deranged expression of specific adipose lncRNAs may participate in the sequelae of obesity, such as hypertension and type 2 diabetes [46,47], being also related to the deleterious consequences that follow over-nutrition and meta-inflammation [[48], [49], [50]]. Moreover, the study of adipocyte-related lncRNAs in obese patients elective for bariatric surgery has shed light on their association with metabolic determinants of an obese phenotype [22,23,51]. Yet, these studies are limited in number, and tend to prioritize transcripts that are opposite to weight loss, thus positively related to obese outcomes. In the current work, we endeavoured to characterize linc-GALNTL6-4 as an unprecedented biomarker of adipose tissue physiological conditions inverse to an obese phenotype. The clinical weight of this unique lncRNA was first reported in hepatocellular carcinoma [52] and gastric cancer patients with low-expressed linc-GALNTL6-4 [53], which indicated the worst prognosis and survival rates. In fat cells, linc-GALNTL6-4 may preserve adipocyte function, and thus be of use as a therapeutic candidate in the field of obesity and related diseases. Accordingly, the scrutiny of linc-GALNTL6-4 dynamics in adipose biopsies from obese subjects revealed overall decreased levels apparently driven by the inflammatory component of obesity, the levels being restored when this condition was solved upon weight loss. To study if these fluctuations have functional relevance, we surveyed the specific contribution of linc-GALNTL6-4 to adipocyte commitment. Accordingly, while its genetic loss-of-function in adipocyte progenitors blunted the adipogenic course, increased linc-GALNTL6-4 bolstered their adipogenic transformation, thus serving as a positive rheostat for adipocyte function.

As increased linc-GALNTL6-4 in differentiating adipocytes was equally located in both the nucleus and cytoplasm, we hypothesised that its functional activity was mainly conducted through the regulation of gene expression patterns relevant to the adipocyte phenotype. To assess the impacts of linc-GALNTL6-4 on global transcriptomes, these were studied in genetic models of loss and gain-of-function. In the context of linc-GALNTL6-4 knock-down, the number of genes affected pictured its significance to the core-essence of human adipocytes. Indeed, the gene expression patterns in treated cells were suggestive of multiple altered states, ranging from inflammation to compromised cell cycle. While the activation of inflammatory cascades is enlisted amongst the most prominent features of an obese phenotype [54], it has only recently been shown that human adipocytes can activate an endoreplicative program as a relevant part of a cellular response to their microenvironment, enabling further adaptation to the hyperplastic expansion of fat depots [55]. In the context of sustained inflammation, the impact of cytokines may constitute the molecular basis of cell-cycle arrest and establishment of senescent-associated molecular patterns [56,57], thus contributing to the pathological consequences of obesity through disablement of functional adipocytes [58]. Our results identify reduction of linc-GALNTL6-4 as an important factor underlying cell cycle defects, as human adipocytes challenged with si-RNAs against this lncRNA displayed compromised expression of genes driving the cell cycle (e.g., *E2F2, E2F8, MAD2L1,* and *SMOC2*), while contributing to the enrichment of factors associated with cell cycle arrest (*BMP4, BRINP2,* and *RCSD1*, amongst others). Puzzlingly, linc-GALNTL6-4 overexpression had little impact on gene expression patterns, and did not significantly activate adipocyte transcriptional networks. The interpretation of this is that, while overexpression and deletion may prompt opposite phenotypes, the overrepresentation of this lncRNA might result in the activation of counteracting processes to block its activity, either due to expression of a rate-limiting inhibitor, or by modifying the amount of putative additional factors required for displaying its function.

Reinforcing the apparent impact of linc-GALNTL6-4 on transcriptomes, LoF and GoF adipocytes were subjected to quantitative lipidomics, which depicted distinct changes in lipid composition. Notably, the LoF and GoF in MA, as well as MCM treatment of MA and the GoF in adipocyte precursor cells, caused opposite outcomes with regard to the lipid landscape. The lipidome of adipocytes lacking linc-GALNTL6-4 displayed an overall reducing trend in many lipid classes (SM, PS, PE P, PE, PC, LPE and CE), while PG, HexCer and TG were elevated, partially matching the lipid pattern changes observed in inflamed adipocytes. It is noteworthy that some PE plasmalogens (e.g., PE P-16:0/22:4, PE P 18:1/22:3, and PE-P18:1/22:4) were significantly reduced and increased upon linc-GALNTL6-4 knock-down and overexpression, respectively. This indicates an alteration in the synthesis of plasmalogens and, accordingly, in the function of peroxisomes with a central role in the biogenesis of these ether lipids [59]. Of note, the reduction in plasmalogens may affect the mitochondria, the function of which is compromised in obesity [60,61], as these lipids play important roles in mitochondrial fission and proliferation [62]. Peroxisomal lipid synthesis and plasmalogens are suggested to orchestrate obesity and insulin resistance [63] – thus, their reduction in adipocytes with linc-GALNTL6-4 knocked-down is consistent with an anti-obesogenic function of this lncRNA, a notion also supported by the observed increase of TGs as a class upon the LoF, and the rise of multiple TG species containing long-chain fatty acids (≥TG 52) in MCM-inflamed adipocytes. Another interesting detail from the lipidomics data is a reduction of the PC species PC30:0, PC32:0 and PC34:2 upon linc-GALNTL6-4 depletion (but not in inflamed adipocytes), which most likely have palmitate (16:0) as one of their fatty acyl chains. As the TGs are elevated as a class, their synthesis is likely enhanced (the other obvious possibility being their reduced hydrolysis); we find it plausible that palmitate is in cells subjected to linc-GALNTL6-4 knock-down preferably channelled to neutral lipid synthesis, and less to the PCs, providing a putative explanation to the altered PC molecular species distribution. To conclude, the observed changes in the adipocyte lipidome upon manipulations of linc-GALNTL6-4 support our notion that this lncRNA acts anti-obesogenic and its reduced expression in obese adipose tissue promotes through multiple mechanisms the metabolic dysregulations characteristic of obesity.

To map the molecular environment of our lncRNA candidate, we surveyed co-expression networks reconstructed for human adipocytes with high and low linc-GALNTL6-4 expression levels. Such re-wiring among genes and interaction hubs in adipocytes, together with our results in gain and loss-of-function cell models, suggested a consistent impact of linc-GALNTL6-4 on APOC1 expression. This was further confirmed in additional assays, and by querying our human cohorts, in which decreased linc-GALNTL6-4 in obese subjects associated with the onset of hypertriglyceridemia and dysregulation of adipose APOC1 gene expression, enhanced response to fatty acids, and impaired adipose tissue activity. As a matter of fact, previous studies performed in humans [64,65] and mice [66] have connected the effects of increased APOC1 in combined hyperlipidaemia with a more pronounced impact on circulating triglycerides [67,68]. On the other hand, sphingomyelin underrepresentation, together with the enrichment of hexosylceramides paralleling reduced linc-GALNTL6-4 expression, may be related to the catalytic activity of APOC1, which includes the cleavage of sphingomyelins to generate ceramides [69], contributing to some of the most common sequelae of obesity [70]. It should be noted, however, that other mechanisms of action are plausible and cannot be discarded at this point. For instance, the ability of cytoplasmic linc-GALNTL6-4 as a competing endogenous RNA of miR-494 has been described in hepatoma cells [52], preventing the translational repression and messenger RNA decay of certain miR-494 target genes. Notably, miR-494 is linked, not only to the progression of endometrial cancer [71] and metastasis of hepatocellular carcinoma [72], but also to the adipogenic transformation and mitochondrial biogenesis of white and beige adipocytes [73], and thus it has been defined as an “anti-adipogenic” miRNA [74]. As a matter of fact, when uploaded to g:Profiler, interpretation of a list of 627 potential miR-494-3p target genes (TargetScanHuman_8.0) highlighted the regulation of primary metabolic processes (GO:0080090) as the most represented biological pathway (adjusted p-value = 4.98E-22). Noteworthy, 143 (22.8%) of these miR-494-3p target genes were downregulated in our LoF model (5.2% of the genes decreased), while only 90 (14.35%) were significantly increased in adipocytes subjected to the depletion of linc-GALNTL6-4 (2.9% of the sum of genes upregulated). With a z-score test value of −4.42 and p-value<0.00001, these proportions differ significantly, and support to a certain extent the post-transcriptional modulation of adipocyte transcriptomes when there is less cytoplasmic linc-GALNTL6-4 sponging miR-494-3p (Fig. S5a). Obviously, this does not cover for the nuclear presence of this lncRNA, and thus, the observation needs to be interpreted with caution, especially when changes in GoF transcriptomes did not reach the statistical significance (Fig. S5b).

Since the characterization in 1990 of the mouse H19 gene product [75], the number of studies addressing the biological significance of long RNA molecules that do not code for proteins has increased exponentially in almost all fields of life science [76]. There is a keen interest on these RNA species in biomedical research, as lncRNAs may provide biomarkers and attractive therapeutic targets in multiple illnesses, ranging from cancers to cardiometabolic diseases [77,78]. While the implication of adipose-expressed lncRNAs in metabolic disorders enhances the need of additional research, the present study constitutes a resource to guide experimental investigations of the posttranscriptional regulation of lncRNAs expressed in adipose tissues, with impact on the welfare of committed adipocytes. To this end, bariatric surgery is widely recognized because of the achieved significant loss of fat mass and reduction in the inflammatory component of obesity [79]. Given the crucial importance of meta-inflammation, the interest in unravelling genomic alterations and new mediators of energy homeostasis in the context of the obese phenotype is growing exponentially. Here, amongst all the lncRNAs modified upon massive weight loss, we resolved to study the one most robustly upregulated. This novel regulatory element of adipocyte genomes and lipid patterns appears to be compromised by excessive body weight and the inflammatory issues that jointly define the obese phenotype. This may hinder the control exercised by this unique lncRNA on adipocyte *APOC1*, thus compromising adipose tissue function and lipid homeostasis to the point of affecting dyslipidaemia.

## 4 Methods

### 4.1 Clinical cohorts

To identify lncRNAs of relevance in the obesity arena, we reanalysed microarray transcriptomes of adipose tissue before and ∼2-years after surgery-induced weight loss (accession number GSE53378 in the Gene Expression Omnibus (GEO) public repository, http://www.ncbi.nlm.nih.gov/geo/) [20]. The study was performed in an identification sample of 16 morbidly obese women randomly selected from a final sample of 23 (see Supplemental Table S1). Additional fat samples were retrieved and analysed in cross-sectional comparisons. These were obtained during elective surgical procedures (i.e., cholecystectomy, surgery of abdominal hernia and gastric bypass) from SC and OM fat depots (151 paired samples) in an independent cohort of 212 participants (Table S4). In both cohorts 1 and 2, macrobiopsies of human adipose tissue were minced in pieces of ∼150 mg immediately after extraction, then introduced in sterile containers, snap frozen in liquid nitrogen, and stored at minus 80 °C until further processing. For the study of isolated SVC and MA, ∼5 g of adipose tissue were retrieved from 12 obese women, digested, disaggregated and centrifuged, as explained in reference [80]. Human samples were processed following standard operating procedures, and information from subjects included in this study was provided by the FATBANK platform, promoted by the CIBEROBN and coordinated by the Biomedical Research Institute of Girona (IDIBGI) Biobank (Biobank IDIBGI, B.0000872), integrated in the Spanish National Biobanks Network (ISCIII, Madrid). On the other hand, the METabolic Syndrome In Men (METSIM) cohort consists of 10,197 Finnish males with ages between 45 and 73, recruited in the University of Eastern Finland and Kuopio University Hospital, Kuopio, Finland, as described in detail previously [26]. In this study, we analyzed bulk RNA-sequencing (bulk RNA-seq) data of subcutaneous adipose tissue (SAT) from 335 unrelated men [26,27]. The study design was approved by the Ethics Committee of the Northern Savo Hospital District, and all participants gave written informed consent. All research conformed to the principles of the Helsinki Declaration.

### 4.2 Clinical measures

BMI was calculated as weight (in kilograms) divided by height (in meters) squared. Obesity was defined as BMI≥30 kg/m^2^. Percent fat mass was obtained by bioelectrical impedance analysis (Tanita Corporation). Blood pressure was measured in the supine position on the right arm after a 10 min rest. A standard sphygmomanometer of appropriate cuff size was used, and the first and fifth phases were recorded. Serum glucose was assessed in duplicate by the glucose oxidase method in a Beckman Glucose Analyser 2 (Beckman Coulter). Measures of total cholesterol were obtained by the reaction of cholesterol esterase/oxidase/peroxidise on a BM/Hitachi 747 analysis system (Roche), and high (HDL) and low (LDL) density lipoproteins were quantified after precipitation with polyethylene glycol 6000 at a final concentration of 100 g/l. Blood haemoglobin (HbA1c) was measured during routine laboratory tests, and blood triglycerides were quantified by the reaction of glycerol-phosphate-oxidase and peroxidase on a Hitachi 917 Rack Chemistry Analyser (Roche). All participants were requested to withhold alcohol and caffeine during at least 12 h prior the analyses, and were recruited at the Department of Diabetes, Endocrinology and Nutrition and the Department of Surgery of the Hospital “Dr Josep Trueta” of Girona (Spain). All subjects provided written informed consent before entering the study, which was approved by the Ethics Committee for Clinical Investigation (CEIC) of the IDIBGI.

### 4.3 Co-expression analysis

Spearman's correlations for linc-GALNTL6-4 were studied in adipose tissue transcriptomes at the baseline and after weight loss to indicate common genes potentially underlying mechanisms related to its expression. Computed values of correlation provided a list of genes with expression levels significantly associated with adipose linc-GALNTL6-4. Implementation of results from semi-independent samples was assessed to suppress spurious associations from output results when interpreting co-expression datasets that include thousands of variables [81]. These lists of genes were uploaded into the QIAGEN Ingenuity Pathways Analysis (IPA) 8.7 software (http://www.ingenuity.com) and g:Profiler version e110_eg57_p18_4b54a898 [82] to interpret significant associations in the context of enriched pathways and common hallmarks associated with our lncRNA candidate in adipose tissue.

### 4.4 RNA-seq data in METSIM

We aligned previously generated bulk SAT RNA-sequencing data from the METSIM cohort (GSE135134) [26,27] to the GRCh38 genome with GENCODE v42 annotations with STAR v.2.7.10a [83] in a two-pass mode, where the splice junctions identified from the first pass were provided as an additional input to the –sjdbFileChrStartEnd flag in the second pass, to account for novel splice junction sites. Technical metrics and counts at the gene name level were obtained using the CollectRnaSeqMetrics command from Picard Tools v2.13.2 and featureCounts v2.0.2 [84], respectively. We tested the linc-GALNTL6-4 for DE by the continuous outcomes of BMI and fat mass, and the binarized outcomes of obesity and fat mass in SAT bulk RNA sequencing data from the METSIM cohort (n = 335) using limma [85] with the voom normalization method [86]. The continuous outcomes were adjusted for age and quantile normalized, while for the binarized outcomes, we compared expression between individuals with (BMI≥30) and without obesity (BMI<30), and fat mass above or below the median of 20.7%. We first filtered the bulk expression data to retain only expressed genes, with at least 1 count per million mapped reads in at least 10% of the samples, and TMM normalized the filtered expression data. We then modelled gene expression on each of the outcomes, where we included the covariates of batch, RIN, uniquely mapped read percentage, mitochondrial read percentage, median 3’ bias, and intronic base percentage to account for technical variation. After fitting the described model for all expressed genes, we tested for the effect of the outcome on linc-GALNTL6-4 expression.

### 4.5 Cell cultures

Commercially available SC adipocyte progenitor cells from one female donor without (BMI<25 kg/m^2^) and one female donor with (BMI>30 kg/m^2^) obesity (SP-F-1 and SP-F-3, respectively), both of approximately same age (35–40 yrs), were plated in culture and stimulated with adipogenic conditions. Preadipocytes (SP-F-2) from one woman and one man of approximately same age (35–45 years) and BMI (25–30 kg/m^2^) were also used during this research, together with culture and differentiation media as recommended by the distributor (Zen-Bio, Inc.). One additional SP-F-3 from an obese female donor, and several other vials SP-F-2 from one female donor were purchased, cultured, differentiated and treated as explained below. To induce adipogenic conversion, human PA were led to grow into a monolayer in preadipocyte media (PM-1), then incubated with adipocyte differentiation media (DM-2) for 7 days. This media is composed of DMEM/Ham's F-12 (1:1), HEPES, FBS, biotin, pantothenate, insulin, dexamethasone, IBMX, PPARγ agonist, penicillin, streptomycin and amphotericin B. Thereafter, differentiating adipocytes were maintained in adipocyte maintenance (AM-1) media (DM-2 without IBMX and PPARγ agonists) for 7 days. During this process, the shape of preadipocytes evolves from the flattened form to rounded cells containing abundant cytoplasmic lipid droplets (Figure 2F), and are thus considered differentiated, mature adipocytes (∼12th day and thereafter). Beyond day 14th, lipid-filled mature fat cells were maintained in adipocyte basal medium (BM-1), which contains the components necessary to support stability and activity of adipocytes (DMEM/Ham's F-12 (1:1), HEPES, biotin and pantothenate). To check whether stimuli that may compromise adipocyte function led to reciprocal changes in the expression of linc-GALNTL6-4, cultures of human adipocytes were challenged with maintenance media containing 2% macrophage LPS-conditioned media (MCM) or control (macrophage media). To this purpose, the human monocyte cell line THP-1 (ATCC, TIB-202) was first cultured in RPMI 1640 medium containing 10% fetal bovine serum (Gibco, 10270-106), 5 mM glucose, 2 mM L-glutamine, 50 mg/ml Gentamicin (Sigma, G1397), and 20 mM HEPES at 37 °C in a humidified 5% CO_2_ and 95 °C air atmosphere. The type 1 macrophage-like state (M1) was induced by adding to the media 0.162 mM phorbol 12-myristate 13-acetate (Sigma, P8139). After 24 h, plastic-adherent M1 macrophages were washed with Dulbecco's phosphate-buffered saline and incubated with fresh medium without PMA. Then, these cell cultures were treated with 10 ng/ml lipopolysaccharide (Sigma, L4516) for 24 h. The resulting media, rich in macrophage-released cytokines [87], was collected and centrifuged 5 min at 400 g, diluted into adipocyte medium (2%), and used to induce inflammation in MA (Figure 2G). We also investigated the regulation of linc-GALNTL6-4 in adipocytes responding to additional inflammatory stimuli, including 100 ng/ml recombinant TNFα (R&D Systems, #10291-TA) and 0.1 μg/ml LPS (TLR4 agonist), as well as the synthetic double-stranded RNA analogue Poly(I:C) (TLR3) at 20 μg/ml and 1 μg/ml Pam3CSK4 (TLR1/2) (R&D Systems, reference #4287 and #4633, respectively).

### 4.6 FISH

Non-differentiated precursor cells and differentiating adipocytes were rinsed with PBS and fixed in 3.7% formaldehyde for 10 min at room temperature. Cells were then permeabilized with 70% ethanol for 1 h at 4 °C. Stellaris RNA Fluorescence In Situ Hybridization (FISH) probes (LGC Biosearch Technologies), covering the length of the target transcript and conjugated with the Quasar 570 fluorophore, were used together with the Stellaris RNA FISH Hybridization Buffer (SMF-HB1-10). The hybridization media was maintained overnight at 37 °C in a humidified chamber, according to the Stellaris protocol for adherent cells. After washing, coverslips were mounted using the Vectashield Antifade Mounting Medium with DAPI (Vector Laboratories, H-1200-10). Cell imaging was performed using a Nikon A1R HD25 Confocal Microscope, with the 60× oil immersion objective of 1.4 NA. The Nikon's Flagship NIS-Elements Package software was used for image analysis.

### 4.7 Loss-of-function

To accomplish the knockdown of linc-GALNTL6-4 in differentiating adipocytes, we used home-made lentiviral particles coding for oligonucleotides against target sequences located in our lncRNA candidate, or scrambled short hairpin non-targeting, non-silencing (sh-NS) RNAs. These lentiviruses were synthetized in HEK293T epithelial-like cells (ATCC, CRL-3216) after co-transfection of plasmids encoding these sh-RNAs, and a combination of packaging (pCMV-dR8.2 dvpr) and envelope (pCMV-VSV-G) plasmids from Addgene. To this end, we used the LipoD293™ DNA In Vitro Transfection Reagent (SigmaGen Laboratories, SL100668). Cultures of human PA were infected with lentiviral particles (1:1) together with 10 μg/μl of polybrene (Santa Cruz, sc-134220). Twenty-four hours’ post-infection, medium was replaced, and transfected cells were enriched by adding 3 μg/ml puromycin dihydrochloride (Sigma, P8833). Then, adipogenic conversion was induced, and MA at day 14th were harvested or fixed as explained above. In parallel, terminally differentiated, lipid-containing MA were obtained as per normal and cultured in the ectopic presence of small interfering (si-)RNA particles against linc-GALNTL6-4 for 72 h. Two constructs were designed to obtain an efficient LoF, one of them achieving the expected downregulation. The sequences used were: GAGATAAGAGGCTGTAGAA[dT][dT] (sense), and TTCTACAGCCTCTTATCTC[dT][dT] (antisense). The MISSION® siRNA Universal Negative Control #1 (Sigma, SIC001) was used as non-targeting control in all experiments. Transfection of differentiated adipocytes and precursor cells was performed as follows: si-RNA and Lipofectamine RNAiMAX (Invitrogen, 13778-075) were diluted with Opti-MEM I® Reduced Serum Medium (Gibco, 31985-062) and mixed by pipetting afterwards. Complexes were left to incubate for 20 min at room temperature and added drop-wise on adherent cells. The final concentration of Lipofectamine RNAiMAX and si-RNAs in 12-well cell culture plates was 1.6 μl/cm^2^ and 100 nM, respectively, and the final amount of medium per well 1.5 ml. Fat cells were harvested 72 h after transfection.

### 4.8 Gain-of-function

Increased expression of linc-GALNTL6-4 in primary human PA and *in vitro* differentiated MA was performed by means of a customized plasmid. linc-GALNTL6-4 was first obtained and amplified from human liver cDNA, using specific primers with restriction sites for KpnI and FseI (Fwd, 5′-GGTAC∗ CCTTTTGCAGAAGTAGATGTGCG-3’; Rev, 5′-GGCCGG∗ CCATTCAAGTGGTTGTTTTATTGACA-3′). The resulting amplicon was purified and cloned into a modified pCMV6 vector (Origene, PS100001). Then, human PA or already differentiated MA were transfected 1:3 with our plasmid construct or an “empty” plasmid control using the FuGENE® HD Transfection Reagent (Promega).

### 4.9 Oil-Red O staining

Oil-Red O staining (Abcam, ab150678) was used to measure intracellular lipid content in adipocytes. Cells were washed with PBS before being fixed with paraformaldehyde 7% (Sigma, 158127) for 1 h and then dipped in isopropanol 60%. When completely dried, cells were stained for 10 min with filtered 5% Oil-Red O in isopropanol at room temperature and washed four times with distilled water. An inverted microscope was used to take pictures. After completely dried, cells were dipped in 100% isopropanol and incubated for 10 min to elute Oil-Red O. Optical density was measured at 500 nm using a PowerWave™ XS spectrophotometer (BioTek Instruments).

### 4.10 Real time PCR

Total RNA was purified from human adipose tissue and cells using RNeasy Mini Kit (QIAgen, 74104). Fat samples (∼150 μg) and cell monolayers were homogenized in 0.6 mL of QIAzol® Lysis Reagent (QIAgen, 79306). RNA was also purified from the nuclear and cytoplasmic component of differentiated lipid-containing human adipocytes, previously isolated by means of the Cytoplasmic and Nuclear RNA Purification Kit (Norgen Biotek, 21000). After addition of chloroform (0.4 volumes), the homogenate was separated into aqueous and organic phases by centrifugation (15 min at 12,000 g and 4 °C). Then, the upper aqueous RNA-rich phase was isolated and ethanol (1.5 volumes) was added to provide appropriate binding conditions. The sample was applied to the RNeasy® Mini spin column, where molecules of RNA bind to the membrane, while phenols and other compounds are washed away. High quality RNA was finally eluted in 30 μL of RNAse-free water. Total RNA concentrations were assessed with a Nanodrop ND-1000 Spectrophotometer (Thermo Scientific). The integrity was checked with the Nano lab-on-a-chip assay for total eukaryotic RNA on a 2100 Bioanalyzer Instrument (Agilent). For real time PCR purposes, RNA was reverse transcribed to cDNA using the High-Capacity cDNA Archive Kit (Applied Biosystems, 4368814). Inventoried TaqMan primer/probe sets from Applied Biosystems (Table S10) were used for gene expression, by using the LightCycler® 480 Real-Time PCR Platform (Roche). PCR reactions were performed in a final volume of 7 μl. The cycle program consisted of an initial denaturing cycle of 10 min at 95 °C, and 45 cycles of 15 s denaturizing phase at 92 °C and 1 min annealing and extension phase at 60 °C. Replicates and positive and negative controls were included. By means of the second derivative method [88], crossing points (Cp) values were obtained for each amplification curve. Then, ΔCp value was first calculated by subtracting the Cp value for endogenous controls (cyclophilin A) in each sample from the Cp value for each sample and target gene. Fold changes compared with the housekeeping were determined by calculating 2^-ΔCp^. β-actin and U6 were used as cytoplasmic and nuclear controls, respectively. Quantitative real time-PCR was also performed to roughly estimate the average lncRNA amounts (copies) in our cell systems (Fig. S2). To do so, we conducted a series of dilutions of our customized plasmid coding for linc-GALNTL6-4, taken as the template to generate a curve-based *in vitro* benchmark system. The copy number of the diluted plasmid (and, thus, linc-GALNTL6-4) was calculated by means of the DNA/RNA Copy Number Calculator (http://endmemo.com/bio/dnacopynum.php), determined according to the plasmid amount (ng) and sequence length (5,200 bp) following the formula below. A standard curve was generated to establish the relationship between the number of linc-GALNTL6-4 copies and Ct values. Then, the RNA collected from a stable amount of cells was subjected to reverse transcription and real time-PCR to obtain the respective linc-GALNTL6-4 Ct values.

$$Number\;of\;copies=\frac{Amount\;\left(ng\right)\times 6.022\times 10^{23}}{Length\;\left(bp\right)\times 1\times 10^{9}\left(\frac{ng}{g}\right)\times 650\;\left(\frac{g}{mol}\right)}$$

### 4.11 RNA-seq

We performed deep RNA sequencing (RNA-seq) in white adipocytes challenged by the si-RNA-mediated silencing of linc-GALNTL6-4 during the last 72 h of terminal differentiation. The library was prepared using poly(A) enrichment, with an average paired-end coverage of 45M read pairs (range 38–49M read pairs) in an NextSeq™ 550 System (Illumina). To investigate the landscape of genes directly regulated by our lncRNA candidate, raw sequencing reads in the fastq files were mapped with STAR version 2.7.1a [83] Gencode release 36, based on the GRCh38.p13 reference genome and the corresponding GTF file. The table of counts was obtained with featureCounts function in the package subread, version 1.6.4 [84]. The differential gene expression analysis (DEG) was assessed with voom + limma in the LIMMA package version 3.46.0 [89] using the R computing software version 4.0.3. Genes having less than 10 counts in at least 4 samples were excluded from the analysis. Raw library size differences between samples were treated with the weighted “trimmed mean method” TMM [90] implemented in the edgeR package [91]. The normalized counts were used in order to make unsupervised analysis, Principal Component Analysis (PCA) and sample clustering. For the differential expression analysis, read counts were converted to log_2_-counts-per-million (logCPM), and the mean-variance relationship was modelled with precision weights using voom approach in the LIMMA package. Pre-Ranked Gene Set Enrichment Analysis (GSEA) [37] implemented in the clusterProfiler [92] package version 3.18.0 was used in order to retrieve enriched functional pathways. Functional annotation was obtained based on the enrichment of genes belonging to gene set collections of the Molecular Signatures Database (MSigDB). The collection used in this project was Hallmark, which summarizes specific well-defined biological states or processes and display coherent expression [93]. Normalized enrichment scores (NES, the enrichment score for the gene set after being normalized across gene sets) and adjusted false discovery rate (FDR) p-values were retrieved and represented in plots. In addition, Metascape (https://www.metascape.org) [94] and Reactome (https://www.reactome.org) [95] were used to create an interactome and enrichment pathway analysis to complement our GSEA results. The results obtained were ranked based on their adjusted p-values and top biological pathways represented in two independent radar plots, including both the number of DE genes for each pathway and their respective –Log_10_ p-values.

### 4.12 Microarray

Gene expression profiling of engineered human adipocytes with increased expressions of linc-GALNTL6-4 was performed using GeneChip® Human Transcriptome Array 2.0 (Affymetrix). The R (version 3.6.0) programming environment (https://www.R-project.org/) was used together with different Comprehensive R Archive Network and Bioconductor packages (http://cran.r-project.org/) [96]. After quality control of raw data, samples were background corrected, quantile-normalized, and summarized to a gene-level using the robust multi-chip average (RMA) [97], obtaining a total of 20,893 transcripts. Clustering methods were used and any possible batch effect was discarded. An empirical Bayes moderated t-statistics model (LIMMA) [89] was built to detect DE genes between the studied conditions. Correction for multiple comparisons was performed using FDR [98], and adjusted p-values were also obtained. GSEA (Hallmark) was also performed with lists of probes showing significant changes between treated and control cells. For this, p-value scores of 0.05 were set as a threshold.

### 4.13 Quantitative lipidomics

Lipid purification, direct flow injection analysis (FIA), and mass spectrometry-based detection were conducted at the Lipidomics Lab Regensburg. For quantitative lipidomics, internal standards were added prior to lipid extraction. An amount of 100 μg protein was processed according to the protocol by Bligh and Dyer [99]. The analysis of lipids was performed by direct FIA using a triple quadrupole mass spectrometer (FIA-MS/MS) and a high-resolution hybrid quadrupole-Orbitrap mass spectrometer (FIA-FTMS). FIA-MS/MS was performed in positive ion mode using the analytical setup and strategy described previously [100,101]. A fragment ion of *m*/*z* 184 was used for lysophosphatidylcholines (LPC) [102]. The following neutral losses were applied: Phosphatidylethanolamine (PE) and lysophosphatidylethanolamine (LPE) 141, phosphatidylserine (PS) 185, phosphatidylglycerol (PG) 189 and phosphatidylinositol (PI) 277 [103]. Sphingosine based ceramides (Cer) and hexosylceramides (Hex-Cer) were analysed using a fragment ion of *m*/*z* 264 [104]. PE-based plasmalogens (PE P) were analysed according to the principles described by Zemski-Berry [105]. Glycerophospholipid species annotation was based on the assumption of even numbered carbon chains only. A detailed description of the FIA-FTMS method was published recently [106,107]. Triglycerides (TG), diglycerides (DG) and cholesterol esters (CE) were recorded in positive ion mode *m*/*z* 500–1000 as [M + NH4]^+^ at a target resolution of 140,000 (at 200 *m*/*z*). CE species were corrected for their species-specific response [10]. Phosphatidylcholines (PC), PC ether (PC O) and sphingomyelins (SM) were analysed in negative ion mode *m*/*z* 520–960 as [M + HCOO]^-^ at the same resolution setting. Analysis of free cholesterol (FC) was performed (after derivatization with acetyl chloride [3]) by multiplexed acquisition (MSX) of the [M + NH4]^+^ of FC and the deuterated internal standard (FC[D7]) [108].

### 4.14 Biochemistry

APOC1 was assessed in cell media using the Human APOC1/Apolipoprotein C–I ELISA Kit (Millipore, RAB0611), an enzyme-linked immunosorbent assays. Triglycerides were measured in cell media by means of the Triglyceride Quantification Kit (Sigma, MAK266), which relies in a colorimetric reaction following the oxidation of glycerol, once triglycerides are broken down into their two principal components (fatty acids and glycerol) by hydrolysis.

### 4.15 WGCNA

Taking into account the transcriptomic landscape of *ex vivo* isolated adipocytes [30], we categorised 22 samples into high (>75%) and low (<25% quantile) linc-GALNTL6-4 expressions, each comprising 5 transcriptomes. A total of 2,516 genes, representing the top 10% of the genome-wide variance, were utilized for hierarchical clustering. One sample was excluded due to poor performance in the clustering process. The remaining 9 samples underwent Weighted Correlation Network Analysis (WGCNA). A soft threshold (K = 6) was applied to determine gene relationships and co-expression networks. After clustering these gene co-expression networks into 8 non-related modules, a linear regression model was employed to estimate the correlation between modules and linc-GALNTL6-4 expression values. Given their rank in the topological overlap matrix (TOM), 20 hub genes were identified for network construction. The R package ‘igraph’ (https://cran.r-project.org/web/packages/igraph/citation.html) and Cytoscape [109] were used to develop the plots embedded in Figure 5.

### 4.16 Enrichment pathway analysis

To interpret biological functions in each module exhibiting significant relationship with linc-GALNTL6-4 expression, pathway enrichment analysis was performed using the genes included in each module [110]. Within each module, the relationship across genes was assessed based on their significance for weight and intramodular connectivity. The R clusterProfiler 4.0 package [92] was employed in conjunction with the KEGG [111], GO [112], Hallmark, and curated gene sets from the MSigDB [93] to predict the function of the gene list of interest. The gene list in ME “brown” underwent over-representation analysis, and the DE genes were utilized for enrichment analysis. Enriched pathways with an adjusted Benjamini-Hochberg p-value<0.05 were considered statistically significant.

### 4.17 Other statistical analyses

Data are expressed as mean ± standard deviation (S.D.) or standard error of the mean (S.E.M.). Statistical analysis was performed using SPSS (IBM Analytics) or GraphPad Prism, version 8.0 (GraphPad Software). Sample sizes (n) are indicated on each figure legend and provided as supplemental tables, and represent values assessed in individuals and biological replicates. Significance was determined by ANOVA (post-hoc Bonferroni) to assess the significance of dynamic changes in linc-GALNTL6-4 levels during adipogenesis, or by two-sided Student's t-test, in comparisons between two independent groups. Differences between paired samples were compared by paired Student's t-test. Spearman's test was employed in correlation analyses. P values of less than 0.05 were considered significant.

### 4.18 Data availability

The data that support the findings of this study has been deposited in public data repositories, or is presented as Supplementary Information with the manuscript. The transcriptomic data generated have been deposited in the GEO repository under accession codes GSE218896 (RNA-seq) and GSE218895 (microarray). These SubSeries constitute the SuperSerie with accession number GSE218897. Additional datasets used during this research are publicly available under the following accession codes: GSE53378 [20] and GSE199063 [24], GSE135134 [[25], [26], [27]], GSE135776 [30], and GSE115020 [31,32]. The source lipidomic data underlying the plots contained in this article was also uploaded to Figshare (https://doi.org/10.6084/m9.figshare.25541005). The raw data deposited in Figshare includes lipid class and species abundance in our lipidomes, as assessed in preadipocytes (PA) and mature adipocytes (MA), with defective (LoF) or increased (GoF) linc-GALNTL6-4, and MCM-inflamed MA versus MA under normal conditions.

## CRediT authorship contribution statement

**Aina Lluch:** Data curation, Formal analysis, Investigation, Methodology. **Jèssica Latorre:** Conceptualization, Data curation, Formal analysis, Investigation, Methodology, Writing – original draft. **Núria Oliveras-Cañellas:** Investigation. **Ana Fernández-Sánchez:** Investigation. **José M. Moreno-Navarrete:** Conceptualization, Investigation, Methodology. **Anna Castells-Nobau:** Data curation, Formal analysis, Investigation. **Ferran Comas:** Investigation. **Maria Buxò:** Data curation, Methodology. **José I. Rodríguez-Hermosa:** Resources. **María Ballester:** Data curation, Formal analysis, Software. **Isabel Espadas:** Data curation, Formal analysis, Investigation, Methodology, Software. **Alejandro Martín-Montalvo:** Data curation, Formal analysis, Investigation, Methodology, Software. **Birong Zhang:** Data curation, Formal analysis, Software. **You Zhou:** Data curation, Formal analysis, Investigation, Methodology, Software. **Ralph Burkhardt:** Investigation, Resources, Supervision. **Marcus Höring:** Data curation, Formal analysis, Investigation, Methodology, Resources, Software. **Gerhard Liebisch:** Conceptualization, Data curation, Formal analysis, Investigation, Methodology, Project administration, Software, Supervision. **Ainara Castellanos-Rubio:** Resources. **Izortze Santin:** Conceptualization, Resources. **Asha Kar:** Investigation. **Markku Laakso:** Investigation. **Päivi Pajukanta:** Conceptualization, Data curation, Resources, Supervision. **Vesa M. Olkkonen:** Conceptualization, Data curation, Formal analysis, Investigation, Supervision, Writing – review & editing. **José M. Fernández-Real:** Conceptualization, Resources, Supervision, Writing – review & editing. **Francisco J. Ortega:** Conceptualization, Data curation, Formal analysis, Funding acquisition, Investigation, Methodology, Project administration, Resources, Software, Supervision, Validation, Visualization, Writing – original draft, Writing – review & editing.

## Declaration of competing interest

The authors declare that they have no known competing financial interests or personal relationships that could have appeared to influence the work reported in this paper.

## Data Availability

The data that support the findings of this study has been deposited in public data repositories, or is presented as Supplementary Information with the manuscript. Gene Expression Omnibus GEOA new adipocyte-specific long non-coding RNA engages impaired fatty acid sensing and dyslipidaemia in obese subjects (Original data) Figsharehttps://doi.org/10.6084/m9.figshare.25541005 (Original data)

## Appendix A Supplementary data

Figure S1 **(a)** Bulk tissue-specific mRNA expression level measurements, in transcripts per million (TPM), for linc-GALNTL6-4. **(b)** The heatmap shows correlation values between linc-GALNTL6-4 (ENSG00000250266) and adipocyte (AC), adipose progenitor (AP), mesothelial cell (MesoC), endothelial cell (EC), smooth muscle cell (SMC), macrophage (MC), neutrophil (NP), mast cell (MastC), T-cell (TC), and plasma cell (PlasC) reference transcripts assessed in SC and OM (visceral) adipose tissues of men and women in reference [28]. The colours are based on the minimum and maximum scores: the lowest score is the darkest blue, and the highest score is inked in the darkest red, while any shade in between is therefore relative to these scores. In this integrative analysis of human adipose tissue RNA-seq data, ‘Cell enriched’ stands for depot and sex-specific cell-type-enriched coding and non-coding transcripts surpassing a designated correlation threshold. Figure S1

Figure S2 **(a)** We generated a standard curve-based *in vitro* benchmark system to assess the relationship between the number of copies of linc-GALNTL6-4 and real time-PCR Ct values. **(b)** Estimated number of linc-GALNTL6-4 copies in each of the datasets represented in Figure 2F. Dots show results for each biological replicate (wells of the same 12-well plate). Statistical significance was assessed by ANOVA (post-hoc Bonferroni's multiple comparisons test) to assess the significance of dynamic changes in linc-GALNTL6-4 levels during adipogenesis. ns, not significant; ∗∗p < 0.001. On the other hand, the meta-analysis of publicly available transcriptomic studies targeting the differentiation and phenotype of human adipocytes in reference **(c)** [31] and **(d)** [32] confirmed the enrichment of linc-GALNTL6-4. MAAC, membrane mature adipocyte aggregate cultures; wk, weeks; PA, preadipocytes; SD, standard deviation; SE, standard error. Figure S2

Figure S3 **(a)** When MA were challenged with MCM and compared to control (macrophage media without LPS), **(b)** lipidomics indicated an overall decrease in many lipid species, similar to what happened in our linc-GALNTL6-4 LoF model (Figure 3I–K) and opposite to the GoF (Figure 4K–M), **(c)** and especially significant in the case of some sphingolipids (Cer and SM), storage lipids (decreased DG but increased TG), and structural lipids (PC, PE, PI and PS). On the other hand, **(d)** we conducted genetic linc-GALNTL6-4 GoF in PA, and performed quantitative lipidomics, which showed **(e)** an overall impact on lipid profiles, including **(f)** significant modulation of key lipid species. Dots over column mean and error bars show results for each biological replicate (wells of the same 12-well plate). Statistical significance was assessed by two-tailed Student t-test. ∗p < 0.05, ∗∗p < 0.001. Figure S3

Figure S4 Box plots (median, 25th and 75th percentiles, and maximum and minimum values) show total lipid species content (nmol/mg protein), as determined in adipocytes following **(a)** linc-GALNTL6-4 LoF, **(b)** treatment of MCM, and **(c)** linc-GALNTL6-4 GoF, and **(d)**, in preadipocytes (PA), linc-GALNTL6-4 GoF. Absolute values assessed in their respective controls (i.e., non-silencing RNAs; non-stimulated macrophages media; and “empty” plasmids) are shown for each lipid class and experiment. Bar plots (mean and S.E.M.) illustrate the extent of lncRNA target knockdown and overexpression in each dataset. Dots show results for each biological replicate (wells of the same 12-well plate). Statistical significance was assessed by two-tailed Student t-test. ∗∗p < 0.001. Figure S4

Figure S5 Venn diagrams showing reciprocal coincidences amongst potential miR-494-3p target genes (according to TargetScanHuman_8.0) and genes up and down-regulated in our experiments of linc-GALNTL6-4 **(a)** loss (LoF) and **(b)** gain (GoF) of function. A two proportion z-test (or z-interval) score was used to know whether the number of miR-494-3p target genes down and up regulated in each experiment differed significantly (p < 0.05). Figure S5
