# Supplementary material for: A novel long non-coding RNA connects obesity to impaired adipocyte function
Source: Mol Metab. 2024 Oct 1;90:102040. doi: 10.1016/j.molmet.2024.102040 (PMC11544081; doi:10.1016/j.molmet.2024.102040)
Supplement: Figure S1 — (a) Bulk tissue-specific mRNA expression level measurements, in transcripts per million (TPM), for linc-GALNTL6-4. (b) The heatmap shows correlation values between linc-GALNTL6-4 (ENSG00000250266) and adipocyte (AC), adipose progenitor (AP), mesothelial cell (MesoC), endothelial cell (EC), smooth muscle cell (SMC), macrophage (MC), neutrophil (NP), mast cell (MastC), T-cell (TC), and plasma cell (PlasC) reference transcripts assessed in SC and OM (visceral) adipose tissues of men and women in reference [28]. The colours are based on the minimum and maximum scores: the lowest score is the darkest blue, and the highest score is inked in the darkest red, while any shade in between is therefore relative to these scores. In this integrative analysis of human adipose tissue RNA-seq data, ‘Cell enriched’ stands for depot and sex-specific cell-type-enriched coding and non-coding transcripts surpassing a designated correlation threshold. [file mmc1.pdf]

a

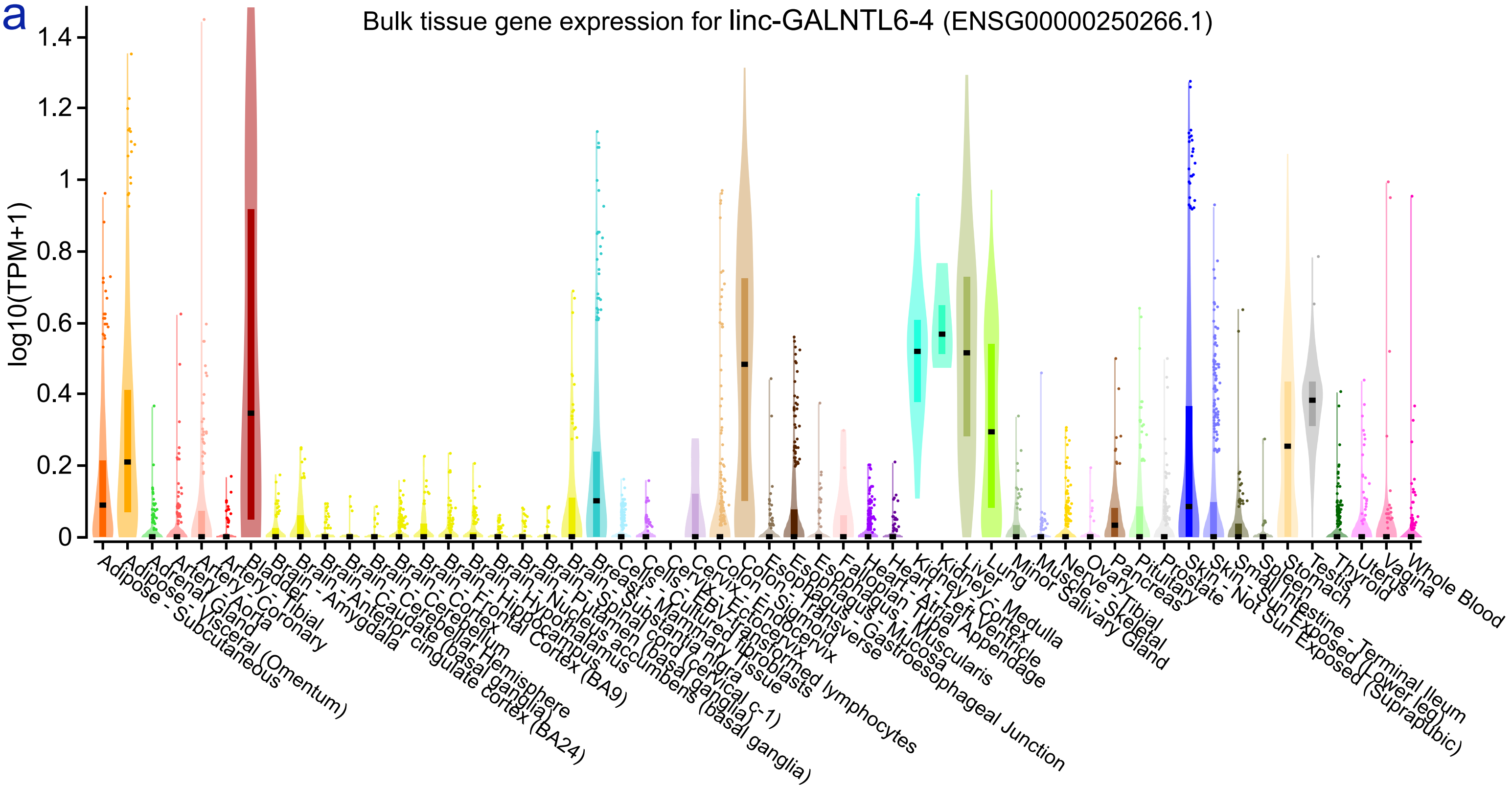

b

Mean expression levels and correlation between linc-GALNTL6-4 and cell-specific reference transcripts

linc-GALNTL6-4	Mean TPM	TPM<0.1 (%)	AC	AP	MesoC	EC	SMC	MC	NP	MastC	TC	PlasC	Cell enriched
SC women	0.49	34.43	0.29	-0.42	---	-0.06	-0.19	-0.13	---	-0.19	-0.20	-0.09	No
SC men	0.47	36.64	0.35	-0.27	---	-0.12	-0.09	-0.03	---	-0.09	-0.14	-0.12	No
OM women	1.28	19.39	0.48	-0.28	-0.12	-0.24	-0.02	-0.06	-0.08	-0.11	-0.04	-0.09	No
OM men	1.40	17.96	0.53	-0.26	0.04	-0.29	-0.09	-0.11	-0.10	-0.10	-0.23	-0.14	AC
