# Supplementary figures and images for: A novel long non-coding RNA connects obesity to impaired adipocyte function
Source: Mol Metab. 2024 Oct 1;90:102040. doi: 10.1016/j.molmet.2024.102040 (PMC11544081; doi:10.1016/j.molmet.2024.102040)

**a**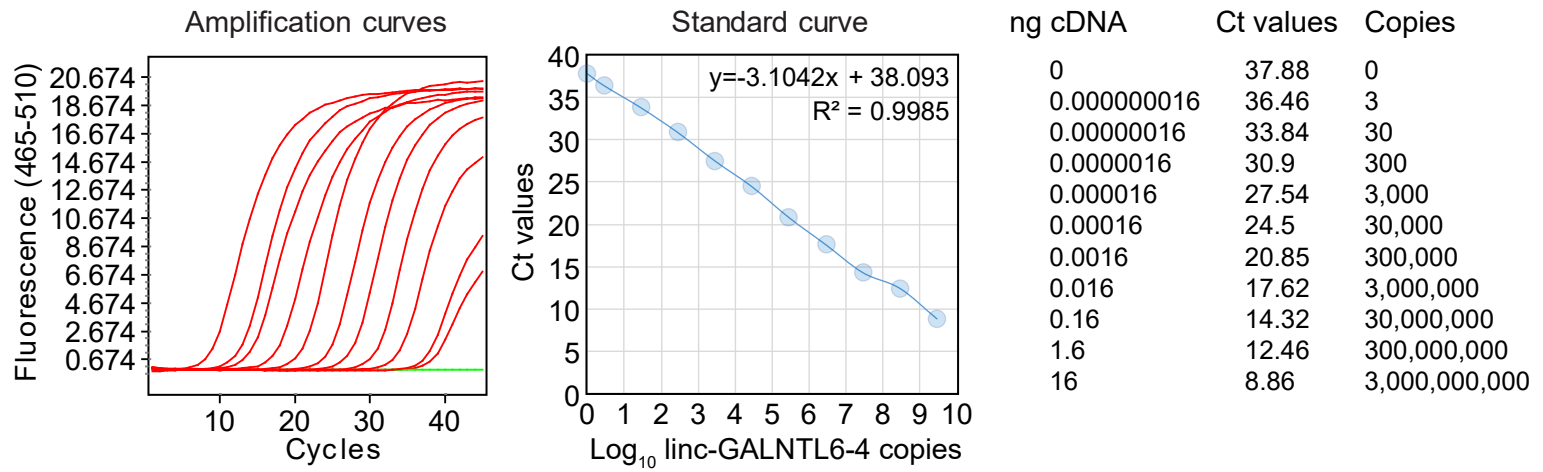**b**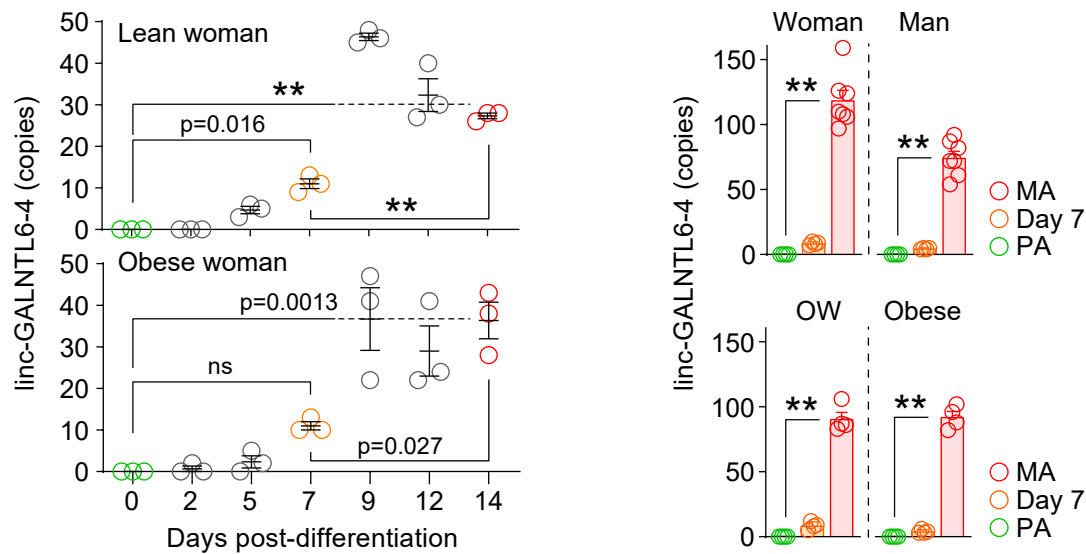**c**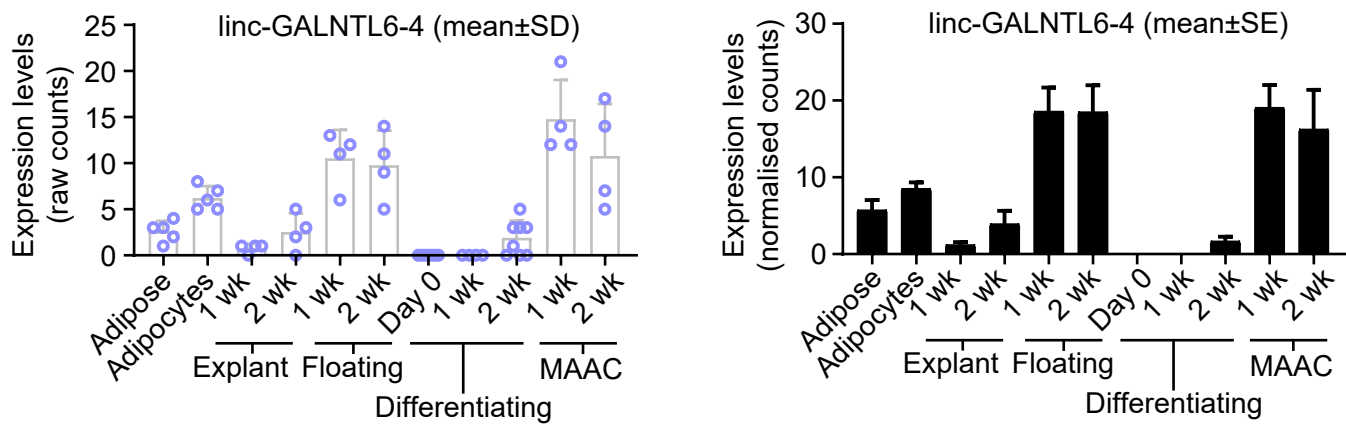**d**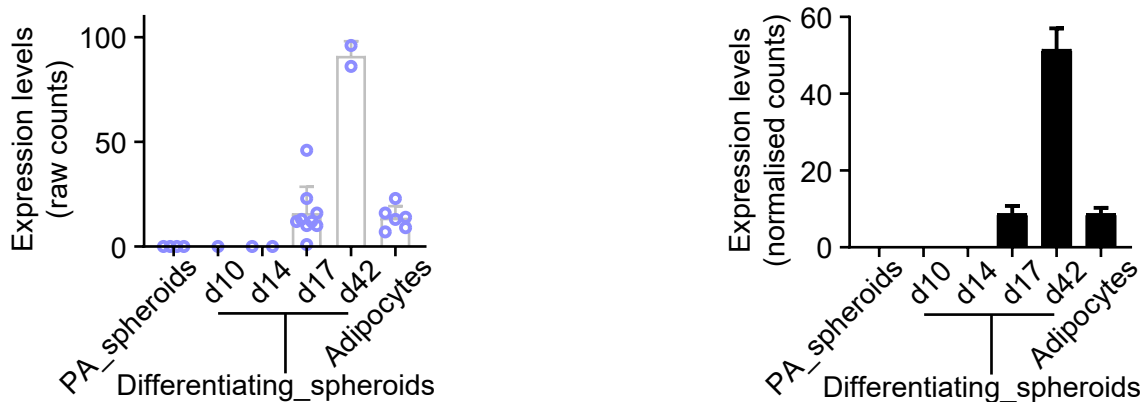

Supplement: Figure S2 — (a) We generated a standard curve-based in vitro benchmark system to assess the relationship between the number of copies of linc-GALNTL6-4 and real time-PCR Ct values. (b) Estimated number of linc-GALNTL6-4 copies in each of the datasets represented in Figure 2F. Dots show results for each biological replicate (wells of the same 12-well plate). Statistical significance was assessed by ANOVA (post-hoc Bonferroni's multiple comparisons test) to assess the significance of dynamic changes in linc-GALNTL6-4 levels during adipogenesis. ns, not significant; ∗∗p < 0.001. On the other hand, the meta-analysis of publicly available transcriptomic studies targeting the differentiation and phenotype of human adipocytes in reference (c) [31] and (d) [32] confirmed the enrichment of linc-GALNTL6-4. MAAC, membrane mature adipocyte aggregate cultures; wk, weeks; PA, preadipocytes; SD, standard deviation; SE, standard error. [file mmc2.pdf]

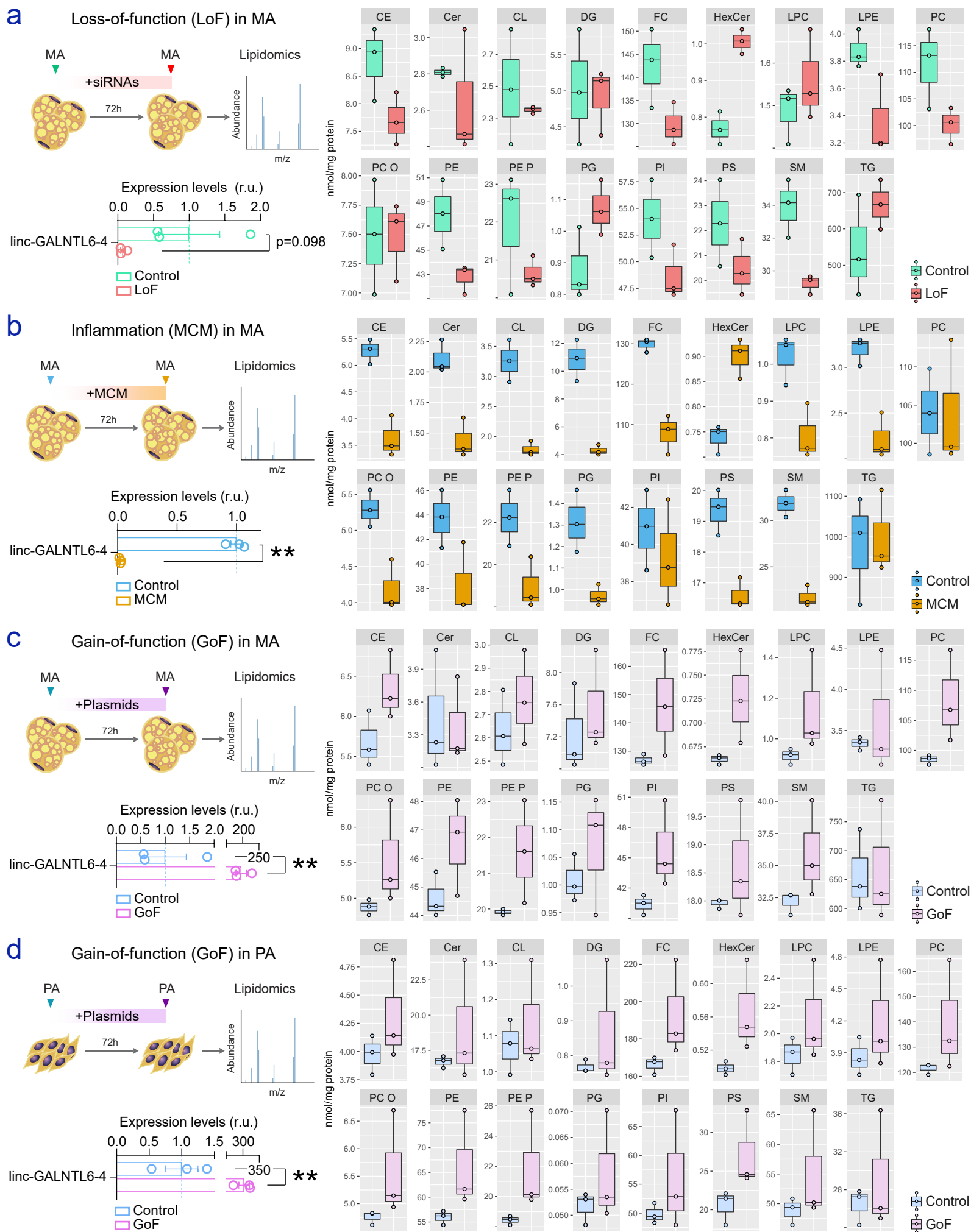

Supplement: Figure S4 — Box plots (median, 25th and 75th percentiles, and maximum and minimum values) show total lipid species content (nmol/mg protein), as determined in adipocytes following (a) linc-GALNTL6-4 LoF, (b) treatment of MCM, and (c) linc-GALNTL6-4 GoF, and (d), in preadipocytes (PA), linc-GALNTL6-4 GoF. Absolute values assessed in their respective controls (i.e., non-silencing RNAs; non-stimulated macrophages media; and “empty” plasmids) are shown for each lipid class and experiment. Bar plots (mean and S.E.M.) illustrate the extent of lncRNA target knockdown and overexpression in each dataset. Dots show results for each biological replicate (wells of the same 12-well plate). Statistical significance was assessed by two-tailed Student t-test. ∗∗p < 0.001. [file mmc4.pdf]
