# Supplementary material for: A novel long non-coding RNA connects obesity to impaired adipocyte function
Source: Mol Metab. 2024 Oct 1;90:102040. doi: 10.1016/j.molmet.2024.102040 (PMC11544081; doi:10.1016/j.molmet.2024.102040)

Figure S3 (LLuch *et al.*)

Quantitative lipidomics in inflamed adipocytes (MCM_MA)

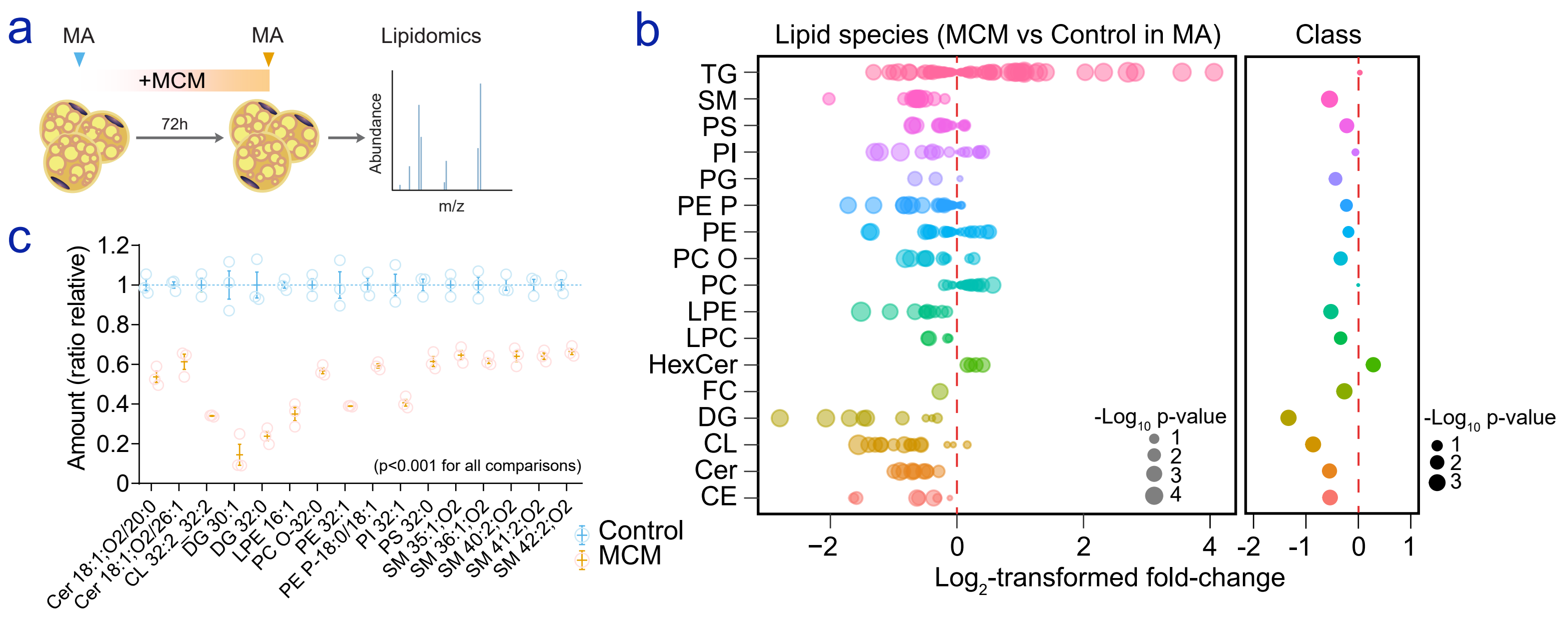

Quantitative lipidomics in human preadipocytes (PA)

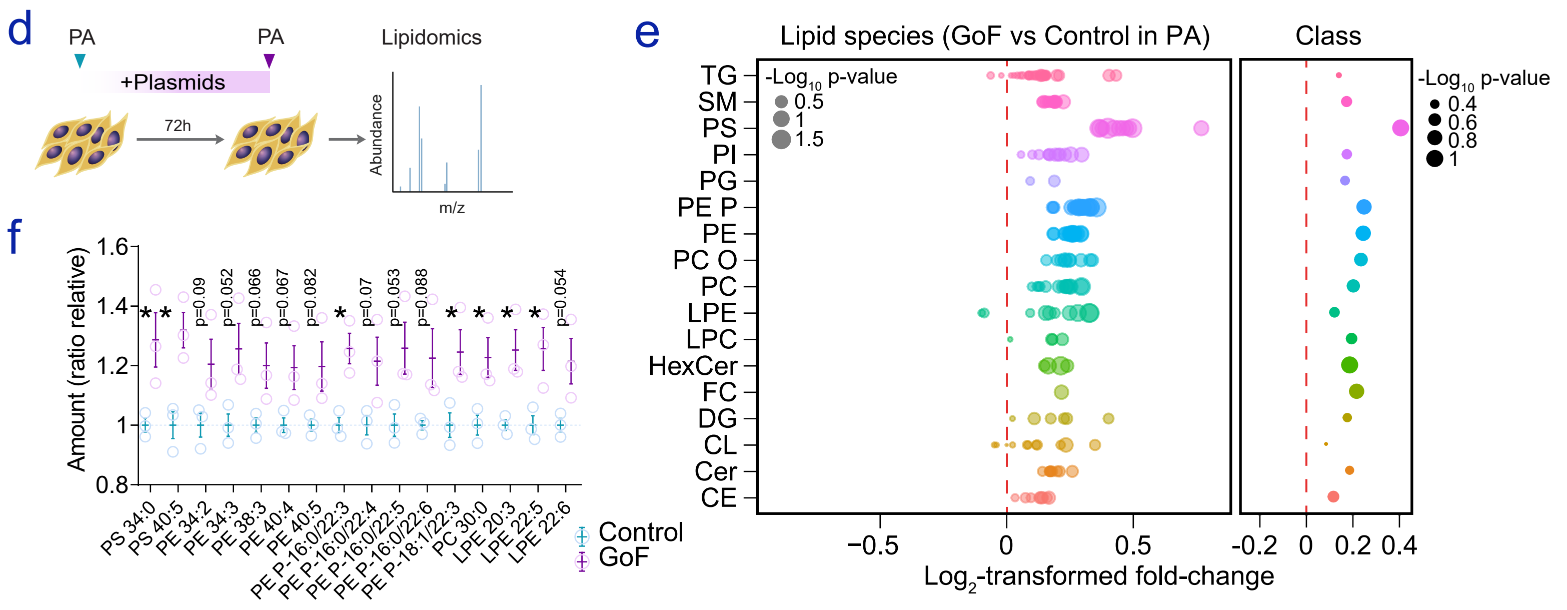

Supplement: Figure S3 — (a) When MA were challenged with MCM and compared to control (macrophage media without LPS), (b) lipidomics indicated an overall decrease in many lipid species, similar to what happened in our linc-GALNTL6-4 LoF model (Figure 3I–K) and opposite to the GoF (Figure 4K–M), (c) and especially significant in the case of some sphingolipids (Cer and SM), storage lipids (decreased DG but increased TG), and structural lipids (PC, PE, PI and PS). On the other hand, (d) we conducted genetic linc-GALNTL6-4 GoF in PA, and performed quantitative lipidomics, which showed (e) an overall impact on lipid profiles, including (f) significant modulation of key lipid species. Dots over column mean and error bars show results for each biological replicate (wells of the same 12-well plate). Statistical significance was assessed by two-tailed Student t-test. ∗p < 0.05, ∗∗p < 0.001. [file mmc3.pdf]
