# Supplementary material for: A novel long non-coding RNA connects obesity to impaired adipocyte function
Source: Mol Metab. 2024 Oct 1;90:102040. doi: 10.1016/j.molmet.2024.102040 (PMC11544081; doi:10.1016/j.molmet.2024.102040)
Supplement: Figure S5 — Venn diagrams showing reciprocal coincidences amongst potential miR-494-3p target genes (according to TargetScanHuman_8.0) and genes up and down-regulated in our experiments of linc-GALNTL6-4 (a) loss (LoF) and (b) gain (GoF) of function. A two proportion z-test (or z-interval) score was used to know whether the number of miR-494-3p target genes down and up regulated in each experiment differed significantly (p < 0.05). [file mmc5.pdf]

a

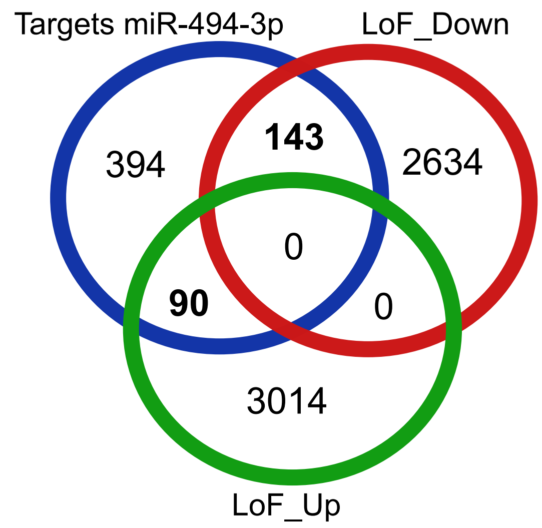

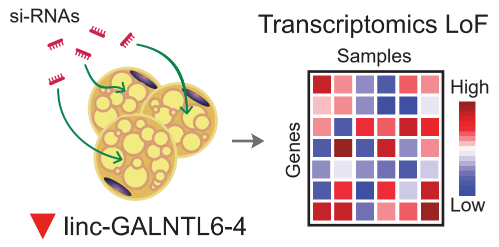

H0: ▼linc-GALNTL6-4 = ▲miR-494-3p function = ▼Target genes
143 out of 2777 (5.15%) genes down-regulated are target genes
90 out of 3104 (2.9%) genes up-regulated are target genes
The value of *z* is -4.4159.
The value of p is < .00001.
The result is **significant** at p < .05.

b

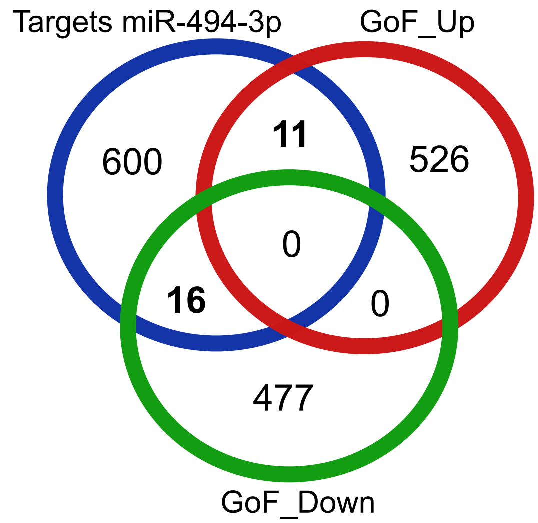

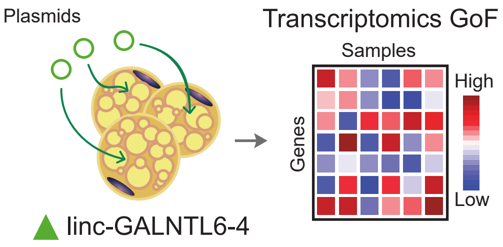

H0: ▲linc-GALNTL6-4 = ▼miR-494-3p function = ▲Target genes
11 out of 537 (2.05%) genes up-regulated are target genes
16 out of 493 (3.25%) genes down-regulated are target genes
The value of *z* is -1.2012.
The value of p is .23014.
The result is **not significant** at p < .05.
