# Supplementary material for: A novel long non-coding RNA connects obesity to impaired adipocyte function
Source: Mol Metab. 2024 Oct 1;90:102040. doi: 10.1016/j.molmet.2024.102040 (PMC11544081; doi:10.1016/j.molmet.2024.102040)
Supplement: Multimedia component 6 [file mmc6.docx]

## SUPPLEMENTARY INFORMATION

**A novel long non-coding RNA connects obesity to impaired adipocyte function**

Aina Lluch^1,2,#^, Jèssica Latorre^1,2,#,^*, Núria Oliveras-Cañellas^1,2^,

Ana Fernández-Sánchez^1^, José M. Moreno-Navarrete^1,2^, Anna Castells-Nobau^1,2^,

Ferran Comas^1^, Maria Buxò^1^, José I. Rodríguez-Hermosa^1,3^, María Ballester^4^,

Isabel Espadas^5^, Alejandro Martin-Montalvo^5,6^, Birong Zhang^7^, You Zhou^7^,

Ralph Burkhardt^8^, Marcus Höring^8^, Gerhard Liebisch^8^, Ainara Castellanos-Rubio^6,9,10^, Izortze Santin^6,9,11^, Asha Kar^12,13^, Markku Laakso^14^, Päivi Pajukanta^12,13,15^,

Vesa M. Olkkonen^16^, José M. Fernández-Real^1,2,3,^*, Francisco J. Ortega^1,2,^*

^1^ Institut d’Investigació Biomèdica de Girona (IDIBGI) – Girona, Spain

^2^ CIBER de la Fisiología de la Obesidad y la Nutrición (CIBEROBN) – Madrid, Spain

^3^ School of Medicine, University of Girona (UdG) – Girona, Spain

^4^ Animal Breeding and Genetics Programme, Institute for Research and Technology in Food and Agriculture (IRTA), Torre Marimon – Caldes de Montbui, Spain

^5^ Centro Andaluz de Biología Molecular y Medicina Regenerativa (CABIMER), Consejo Superior de Investigaciones Científicas (CSIC), University Pablo de Olavide – Seville, Spain

^6^ CIBER de Diabetes y Enfermedades Metabólicas Asociadas (CIBERDEM) – Madrid, Spain

^7^ Systems Immunity Research Institute, Cardiff University – Cardiff, United Kingdom

^8^ Institute of Clinical Chemistry and Laboratory Medicine, University Hospital Regensburg – Regensburg, Germany

^9^ Universidad del País Vasco/Euskal Herriko Unibertsitatea (UPV/EHU) – Bizkaia, Spain

^10^ Ikerbasque, Basque Foundation for Science – Bilbao, Spain

^11^ Instituto de Investigación Sanitaria Biocruces Bizkaia – Bizkaia, Spain

^12^ Bioinformatics Interdepartmental Program, UCLA – Los Angeles (CA), USA

^13^ Department of Human Genetics, David Geffen School of Medicine at UCLA – Los Angeles (CA), USA

^14^ Department of Medicine, University of Eastern Finland and Kuopio University Hospital – Kuopio, Finland

^15^ Institute for Precision Health, David Geffen School of Medicine at UCLA – Los Angeles (CA), USA

^16^ Minerva Foundation Institute for Medical Research, University of Helsinki – Helsinki, Finland

^#^ Equal contribution

*Corresponding author(s):

Francisco J. Ortega, Ph.D.: fortega@idibgi.org

José M. Fernández-Real, M.D., Ph.D.: [jmfreal@idibgi.org](mailto:jmfreal@idibgi.org)

Jèssica Latorre, Ph.D.: [jessica.latorre@uvic.cat](file:///D:\New%20&%20modified%20files\ncRNAs\lncRNA%20in%20AT\lncRNA%20in%20AT%20-%20Rev1\jessica.latorre@uvic.cat)

**Short title:** Adipocyte-specific linc-GALNTL6-4 in obesity.

## SUPPLEMENTAL TABLES

**Table S1.** Anthropometric and biochemical characterization of subjects (all women) included in our longitudinal study. Validation included the Identification subsample.

|  | Identification (Microarray - n=16 paired samples) | | |  | Validation (RT-PCR - n=23 paired samples) | | |
| --- | --- | --- | --- | --- | --- | --- | --- |
|  | Baseline | Post-WL | p-value^a^ |  | Baseline | Post-WL | p-value^a^ |
| Age (years) | 48 ± 10 | 52 ± 10 | <0.0001 |  | 48 ± 9 | 51 ± 9 | <0.0001 |
| BMI (kg/m^2^) | 42.8 ± 4.9 | 27.6 ± 4.7 | <0.0001 |  | 43.1 ± 4.9 | 29.2 ± 5.5 | <0.0001 |
| Fat mass (%) | 57.2 ± 6.7 | 39.7 ± 7.0 | <0.0001 |  | 56.0 ± 7.3 | 40.1 ± 7.4 | <0.0001 |
| SBP (mmHg) | 126.6 ± 10.5 | 128.9 ± 15.9 | 0.579 |  | 128.4 ± 13.6 | 130.7 ± 16.9 | 0.51 |
| DBP (mmHg) | 79.9 ± 8.8 | 74.8 ± 14.4 | 0.223 |  | 79.6 ± 9.8 | 74.2 ± 12.6 | 0.08 |
| Fasting glucose (mg/dl) | 105.3 ± 43.5 | 86.1 ± 16.5 | 0.113 |  | 101.0 ± 35.3 | 87.4 ± 13.5 | 0.079 |
| HbA1c (%) | 5.9 ± 1.6 | 5.3 ± 0.3 | 0.125 |  | 5.8 ± 1.4 | 5.3 ± 0.3 | 0.155 |
| Total cholesterol (mg/dl) | 186.4 ± 38.2 | 189.8 ± 58.1 | 0.792 |  | 182.4 ± 33.1 | 184.7 ± 51.8 | 0.79 |
| HDL cholesterol (mg/dl) | 55.4 ± 14.4 | 75.4 ± 18.5 | <0.0001 |  | 56.0 ± 13.1 | 73.8 ± 22.1 | <0.0001 |
| LDL cholesterol (mg/dl) | 108.3 ± 32.9 | 107.0 ± 31.9 | 0.855 |  | 104.6 ± 28.3 | 100.4 ± 29.7 | 0.406 |
| Fasting triglycerides (mg/dl) | 114.4 ± 43.3 | 83.6 ± 30.7 | 0.024 |  | 109.0 ± 42.5 | 81.4 ± 28.7 | 0.006 |

Data are expressed as mean ± standard deviation (S.D.). WL: weight loss; BMI: body mass index; HbA1c: glycated haemoglobin; H/LDL: high/low-density lipoprotein. ^a^Results post-weight loss vs. baseline were compared by paired *t*-test.

**Table S2.** To identify hub genes, we conducted univariate Spearman regression analyses for linc-GALNTL6-4 and genes expressed in adipose tissue at the baseline (PRE) and ~2 years after weight loss (POST).

| Positive associations | | | | |  | Negative associations | | | | |
| --- | --- | --- | --- | --- | --- | --- | --- | --- | --- | --- |
| Symbol | r-PRE | p-PRE | r-POST | p-POST |  | Symbol | r-PRE | p-PRE | r-POST | p-POST |
| ACADM | 0.51 | 0.0427 | 0.61 | 0.0123 |  | ABCC3 | -0.54 | 0.0304 | -0.72 | 0.0017 |
| ACADS | 0.74 | 0.0010 | 0.52 | 0.0400 |  | ADAMTS2 | -0.51 | 0.0441 | -0.53 | 0.0350 |
| ACO2 | 0.73 | 0.0014 | 0.57 | 0.0210 |  | AJUBA | -0.51 | 0.0427 | -0.55 | 0.0263 |
| ACSS3 | 0.73 | 0.0013 | 0.56 | 0.0244 |  | AKR1C2 | -0.56 | 0.0254 | -0.66 | 0.0052 |
| ADAM5P | 0.54 | 0.0293 | 0.60 | 0.0140 |  | ALCAM | -0.61 | 0.0118 | -0.55 | 0.0283 |
| ADCK3 | 0.74 | 0.0012 | 0.59 | 0.0172 |  | ALOX5 | -0.60 | 0.0134 | -0.54 | 0.0293 |
| AKAP1 | 0.53 | 0.0350 | 0.52 | 0.0374 |  | ANGPTL2 | -0.79 | 0.0003 | -0.68 | 0.0040 |
| ALDH6A1 | 0.68 | 0.0038 | 0.61 | 0.0118 |  | ANXA2 | -0.55 | 0.0273 | -0.62 | 0.0103 |
| ANKRD5 | 0.72 | 0.0016 | 0.52 | 0.0400 |  | ANXA5 | -0.59 | 0.0159 | -0.71 | 0.0022 |
| AZGP1 | 0.72 | 0.0017 | 0.71 | 0.0021 |  | ATXN7 | -0.54 | 0.0326 | -0.53 | 0.0362 |
| AZGP1P1 | 0.52 | 0.0374 | 0.63 | 0.0094 |  | CAPZB | -0.57 | 0.0210 | -0.54 | 0.0293 |
| BAI3 | 0.74 | 0.0009 | 0.54 | 0.0293 |  | CARD6 | -0.69 | 0.0030 | -0.56 | 0.0244 |
| BCKDHB | 0.69 | 0.0029 | 0.71 | 0.0020 |  | CCND2 | -0.72 | 0.0015 | -0.79 | 0.0003 |
| BET1L | 0.62 | 0.0103 | 0.59 | 0.0152 |  | CD84 | -0.50 | 0.0471 | -0.73 | 0.0013 |
| BOK | 0.72 | 0.0016 | 0.51 | 0.0441 |  | CD99 | -0.57 | 0.0218 | -0.62 | 0.0103 |
| CALCRL | 0.56 | 0.0235 | 0.69 | 0.0030 |  | CLIC6 | -0.55 | 0.0283 | -0.86 | 0.0000 |
| CDKN2C | 0.59 | 0.0152 | 0.51 | 0.0413 |  | CMPK1 | -0.60 | 0.0134 | -0.56 | 0.0235 |
| CHODL | 0.53 | 0.0338 | 0.67 | 0.0042 |  | CMTM3 | -0.53 | 0.0338 | -0.61 | 0.0113 |
| CIDEA | 0.57 | 0.0202 | 0.69 | 0.0034 |  | COL12A1 | -0.51 | 0.0413 | -0.62 | 0.0103 |
| COL21A1 | 0.51 | 0.0441 | 0.54 | 0.0326 |  | COL6A1 | -0.51 | 0.0413 | -0.52 | 0.0400 |
| COX4I1 | 0.53 | 0.0350 | 0.71 | 0.0019 |  | CPPED1 | -0.66 | 0.0055 | -0.57 | 0.0210 |
| COX6A1 | 0.61 | 0.0113 | 0.64 | 0.0074 |  | E2F3 | -0.76 | 0.0007 | -0.56 | 0.0227 |
| DCXR | 0.66 | 0.0050 | 0.75 | 0.0008 |  | EGFL6 | -0.59 | 0.0172 | -0.86 | 0.0000 |
| DHRS3 | 0.73 | 0.0013 | 0.60 | 0.0134 |  | ENTPD4 | -0.76 | 0.0007 | -0.52 | 0.0374 |
| ECHDC3 | 0.84 | 0.0001 | 0.58 | 0.0179 |  | FAR2 | -0.57 | 0.0218 | -0.85 | 0.0000 |
| ECHS1 | 0.50 | 0.0486 | 0.65 | 0.0061 |  | FARP1-IT1 | -0.68 | 0.0038 | -0.52 | 0.0387 |
| ECI1 | 0.53 | 0.0350 | 0.66 | 0.0055 |  | FBXO34 | -0.60 | 0.0146 | -0.56 | 0.0254 |
| ELOVL3 | 0.56 | 0.0227 | 0.56 | 0.0235 |  | FLJ14107 | -0.64 | 0.0071 | -0.50 | 0.0486 |
| ESR1 | 0.51 | 0.0427 | 0.61 | 0.0129 |  | FLJ35282 | -0.52 | 0.0387 | -0.57 | 0.0218 |
| FHOD1 | 0.57 | 0.0202 | 0.54 | 0.0315 |  | FMNL1 | -0.59 | 0.0165 | -0.84 | 0.0001 |
| GCDH | 0.71 | 0.0020 | 0.61 | 0.0129 |  | FYN | -0.51 | 0.0456 | -0.50 | 0.0486 |
| GLYCTK | 0.68 | 0.0040 | 0.66 | 0.0050 |  | GALNT13 | -0.56 | 0.0244 | -0.62 | 0.0103 |
| GPD1L | 0.61 | 0.0118 | 0.52 | 0.0387 |  | GLIPR2 | -0.50 | 0.0486 | -0.58 | 0.0187 |
| GPT | 0.60 | 0.0146 | 0.59 | 0.0172 |  | GMIP | -0.55 | 0.0263 | -0.67 | 0.0045 |
| GSDMB | 0.56 | 0.0254 | 0.59 | 0.0172 |  | HDC | -0.66 | 0.0052 | -0.52 | 0.0400 |
| GYS1 | 0.61 | 0.0113 | 0.52 | 0.0374 |  | HDLBP | -0.58 | 0.0187 | -0.63 | 0.0094 |
| HADH | 0.68 | 0.0038 | 0.71 | 0.0021 |  | HSD11B1 | -0.65 | 0.0061 | -0.84 | 0.0001 |
| L2HGDH | 0.53 | 0.0350 | 0.76 | 0.0006 |  | IL18BP | -0.59 | 0.0152 | -0.57 | 0.0210 |
| LDHD | 0.76 | 0.0007 | 0.82 | 0.0001 |  | IL20RB | -0.61 | 0.0118 | -0.69 | 0.0034 |
| LGALS12 | 0.55 | 0.0263 | 0.74 | 0.0011 |  | IQGAP1 | -0.51 | 0.0413 | -0.51 | 0.0441 |
| LPIN1 | 0.56 | 0.0235 | 0.66 | 0.0055 |  | ITGB2 | -0.54 | 0.0293 | -0.57 | 0.0210 |
| MALAT1 | 0.50 | 0.0471 | 0.62 | 0.0108 |  | ITIH5 | -0.64 | 0.0078 | -0.83 | 0.0001 |
| MOGAT1 | 0.61 | 0.0113 | 0.58 | 0.0194 |  | LRRC8E | -0.52 | 0.0387 | -0.52 | 0.0387 |
| MSRB1 | 0.59 | 0.0159 | 0.54 | 0.0304 |  | LRRK1 | -0.54 | 0.0293 | -0.73 | 0.0013 |
| MYO3A | 0.51 | 0.0427 | 0.53 | 0.0350 |  | LUZP1 | -0.59 | 0.0165 | -0.54 | 0.0315 |
| NAPRT1 | 0.67 | 0.0045 | 0.56 | 0.0254 |  | MAP1B | -0.52 | 0.0387 | -0.75 | 0.0008 |
| NDUFB3 | 0.56 | 0.0254 | 0.54 | 0.0293 |  | MASP1 | -0.52 | 0.0400 | -0.70 | 0.0027 |
| NET1 | 0.59 | 0.0165 | 0.50 | 0.0471 |  | MYO5A | -0.54 | 0.0315 | -0.55 | 0.0263 |
| OR13C3 | 0.56 | 0.0227 | 0.57 | 0.0210 |  | NCF4 | -0.53 | 0.0338 | -0.59 | 0.0159 |
| OR4D2 | 0.78 | 0.0004 | 0.53 | 0.0350 |  | NDEL1 | -0.60 | 0.0134 | -0.56 | 0.0244 |
| OR5B12 | 0.62 | 0.0108 | 0.55 | 0.0273 |  | NEK6 | -0.51 | 0.0427 | -0.64 | 0.0074 |
| ORC2 | 0.56 | 0.0254 | 0.53 | 0.0350 |  | NFKB2 | -0.58 | 0.0194 | -0.51 | 0.0427 |
| ORMDL3 | 0.61 | 0.0118 | 0.59 | 0.0172 |  | NRP2 | -0.53 | 0.0350 | -0.73 | 0.0014 |
| PC | 0.55 | 0.0263 | 0.79 | 0.0002 |  | PLAC9 | -0.58 | 0.0194 | -0.80 | 0.0002 |
| PCCA | 0.61 | 0.0129 | 0.62 | 0.0099 |  | PLEKHO1 | -0.59 | 0.0152 | -0.51 | 0.0413 |
| PCK2 | 0.60 | 0.0134 | 0.56 | 0.0235 |  | PLEKHO2 | -0.55 | 0.0263 | -0.53 | 0.0350 |
| PCYT2 | 0.73 | 0.0013 | 0.73 | 0.0013 |  | PMP22 | -0.56 | 0.0254 | -0.60 | 0.0146 |
| PDHX | 0.58 | 0.0194 | 0.68 | 0.0036 |  | PRKCB | -0.51 | 0.0413 | -0.75 | 0.0009 |
| PECR | 0.52 | 0.0387 | 0.69 | 0.0030 |  | PSAP | -0.58 | 0.0194 | -0.71 | 0.0020 |
| PEX11A | 0.66 | 0.0050 | 0.55 | 0.0283 |  | RAB31 | -0.59 | 0.0172 | -0.70 | 0.0027 |
| PEX5L | 0.56 | 0.0235 | 0.56 | 0.0244 |  | RAB7B | -0.61 | 0.0118 | -0.55 | 0.0273 |
| PEX6 | 0.56 | 0.0244 | 0.63 | 0.0090 |  | RAB9A | -0.69 | 0.0030 | -0.57 | 0.0202 |
| PHB2 | 0.52 | 0.0387 | 0.51 | 0.0456 |  | RANBP3L | -0.56 | 0.0227 | -0.51 | 0.0413 |
| PHKA2 | 0.56 | 0.0235 | 0.53 | 0.0338 |  | RFFL | -0.56 | 0.0244 | -0.58 | 0.0179 |
| PLEKHJ1 | 0.64 | 0.0074 | 0.60 | 0.0134 |  | RPS6KA3 | -0.70 | 0.0024 | -0.51 | 0.0413 |
| PLIN5 | 0.56 | 0.0244 | 0.67 | 0.0045 |  | SEC63 | -0.53 | 0.0338 | -0.59 | 0.0159 |
| PPARA | 0.59 | 0.0172 | 0.71 | 0.0022 |  | SERPINB8 | -0.58 | 0.0179 | -0.61 | 0.0113 |
| PTPN3 | 0.55 | 0.0283 | 0.56 | 0.0235 |  | SETBP1 | -0.51 | 0.0456 | -0.68 | 0.0038 |
| PXMP2 | 0.60 | 0.0140 | 0.64 | 0.0078 |  | SIPA1L1 | -0.58 | 0.0179 | -0.73 | 0.0013 |
| PYGM | 0.59 | 0.0159 | 0.59 | 0.0165 |  | SLAMF8 | -0.53 | 0.0338 | -0.61 | 0.0123 |
| RARRES2 | 0.57 | 0.0210 | 0.59 | 0.0152 |  | SLIT3 | -0.57 | 0.0218 | -0.52 | 0.0387 |
| RASL10B | 0.73 | 0.0014 | 0.50 | 0.0486 |  | SNX9 | -0.64 | 0.0074 | -0.50 | 0.0471 |
| SCRN2 | 0.65 | 0.0067 | 0.61 | 0.0113 |  | SRPX2 | -0.68 | 0.0036 | -0.66 | 0.0050 |
| SLC19A3 | 0.79 | 0.0003 | 0.63 | 0.0094 |  | STX3 | -0.66 | 0.0052 | -0.58 | 0.0187 |
| SLC25A1 | 0.61 | 0.0123 | 0.62 | 0.0103 |  | SYNC | -0.71 | 0.0020 | -0.54 | 0.0326 |
| SLC25A6 | 0.64 | 0.0074 | 0.54 | 0.0315 |  | TAOK3 | -0.64 | 0.0074 | -0.79 | 0.0003 |
| SLC2A1 | 0.51 | 0.0427 | 0.58 | 0.0187 |  | TDP1 | -0.55 | 0.0263 | -0.52 | 0.0400 |
| SLC2A4 | 0.66 | 0.0058 | 0.62 | 0.0103 |  | TGFB2 | -0.53 | 0.0362 | -0.55 | 0.0263 |
| SNRPN | 0.57 | 0.0218 | 0.53 | 0.0362 |  | TIAM1 | -0.67 | 0.0045 | -0.58 | 0.0179 |
| SNRPN | 0.55 | 0.0263 | 0.56 | 0.0254 |  | TMEM248 | -0.51 | 0.0427 | -0.55 | 0.0263 |
| SOX6 | 0.61 | 0.0118 | 0.55 | 0.0263 |  | TNFAIP3 | -0.70 | 0.0025 | -0.59 | 0.0159 |
| ST3GAL6 | 0.58 | 0.0194 | 0.62 | 0.0103 |  | TNFAIP6 | -0.63 | 0.0094 | -0.52 | 0.0374 |
| STOX1 | 0.76 | 0.0006 | 0.73 | 0.0014 |  | TRBJ2-2 | -0.55 | 0.0263 | -0.64 | 0.0078 |
| TF | 0.50 | 0.0486 | 0.62 | 0.0099 |  | TUFT1 | -0.59 | 0.0172 | -0.53 | 0.0350 |
| THEM6 | 0.59 | 0.0152 | 0.52 | 0.0374 |  | UBE2H | -0.59 | 0.0152 | -0.51 | 0.0456 |
| TMEM120A | 0.62 | 0.0108 | 0.61 | 0.0118 |  | VPS37C | -0.51 | 0.0413 | -0.60 | 0.0140 |
| TOB2 | 0.69 | 0.0032 | 0.56 | 0.0227 |  | WDR91 | -0.55 | 0.0283 | -0.55 | 0.0263 |
| TRMT44 | 0.52 | 0.0387 | 0.61 | 0.0123 |  | XXYLT1 | -0.52 | 0.0400 | -0.73 | 0.0013 |
| TTLL7 | 0.69 | 0.0029 | 0.50 | 0.0471 |  | ZNF304 | -0.58 | 0.0187 | -0.64 | 0.0071 |
| UQCR11 | 0.57 | 0.0218 | 0.54 | 0.0304 |  |  |  |  |  |  |
| UTS2D | 0.50 | 0.0471 | 0.63 | 0.0090 |  |  |  |  |  |  |
| WDR78 | 0.60 | 0.0146 | 0.57 | 0.0210 |  |  |  |  |  |  |

**Table S3.** Pathways related to linc-GALNTL6-4 (IPA and g:Profiler) according to the gene co-expression network analysis performed in human fat at the baseline and after weight loss.

| Top Canonical Pathways (IPA) | p-value | Overlap | |
| --- | --- | --- | --- |
| Fatty Acid-oxidation I | 1.5E-4 | 13.3% 4/30 | |
| Valine Degradation I | 5.5E-4 | 16.7% 3/18 | |
| Cell Cycle: G1/S Checkpoint Regulation | 3.1E-3 | 6.1% 4/66 | |
| Hepatic Cholestasis | 3.3E-3 | 3.8% 6/158 | |
| Cyclins and Cell Cycle Regulation | 6.2E-3 | 5% 4/80 | |
|  |  | |  |
| Molecular and Cellular Functions (IPA) | p-value range | # Molecules | |
| Energy Production | 2.7E-2 - 1.3E-6 | 9 | |
| Lipid Metabolism | 3.2E-2 - 1.3E-6 | 24 | |
| Small Molecule Biochemistry | 3.2E-2 - 1.3E-6 | 38 | |
| Carbohydrate Metabolism | 2.7E-2 - 8.3E-5 | 19 | |
| Cellular Compromise | 2.7E-2 - 9.3E-5 | 20 | |
|  |  | |  |
| Top Tox Lists (IPA) | p-value | Overlap | |
| Fatty Acid Metabolism | 2.8E-4 | 6.1% 6/98 | |
| Cell Cycle: G1/S Checkpoint Regulation | 3.3E-3 | 6% 4/67 | |
| Hepatic Cholestasis | 3.6E-3 | 3.7% 6/161 | |
| Renal Necrosis/Cell Death | 3.9E-3 | 2.2% 12/538 | |
| Aryl Hydrocarbon Receptor Signaling | 1.2E-2 | 3.4% 5/149 | |

| Pathways depicted by the list of transcripts with direct association with linc-GALNTL6-4 (g:Profiler) | | | | | | | | | | | | |
| --- | --- | --- | --- | --- | --- | --- | --- | --- | --- | --- | --- | --- |
| source | term_name | | term_id | | adj_p_value | | term_size | | query_size | | intersec_size | |
| GO:MF | oxidoreductase activity | | GO:0016491 | | 4.35E-07 | | 753 | | 81 | | 18 | |
| GO:MF | acyl-CoA dehydrogenase activity | | GO:0003995 | | 0.00699696 | | 12 | | 81 | | 3 | |
| GO:MF | delta(3)-delta(2)-enoyl-CoA isomerase activity | | GO:0004165 | | 0.04967026 | | 4 | | 81 | | 2 | |
| GO:BP | organic acid catabolic process | | GO:0016054 | | 4.32E-12 | | 254 | | 81 | | 16 | |
| GO:BP | carboxylic acid metabolic process | | GO:0019752 | | 1.51E-10 | | 948 | | 81 | | 24 | |
| GO:BP | lipid metabolic process | | GO:0006629 | | 2.08E-09 | | 1406 | | 81 | | 27 | |
| GO:BP | carbohydrate metabolic process | | GO:0005975 | | 9.93E-07 | | 573 | | 81 | | 16 | |
| GO:BP | generation of precursor metabolites and energy | | GO:0006091 | | 0.00015913 | | 514 | | 81 | | 13 | |
| GO:BP | response to insulin | | GO:0032868 | | 0.00166697 | | 265 | | 81 | | 9 | |
| GO:BP | branched-chain amino acid metabolic process | | GO:0009081 | | 0.00672536 | | 27 | | 81 | | 4 | |
| GO:BP | triglyceride biosynthetic process | | GO:0019432 | | 0.03043065 | | 39 | | 81 | | 4 | |
| GO:BP | organophosphate metabolic process | | GO:0019637 | | 0.03824277 | | 973 | | 81 | | 14 | |
| GO:BP | regulation of sequestering of triglyceride | | GO:0010889 | | 0.04858542 | | 15 | | 81 | | 3 | |
| GO:CC | mitochondrion | | GO:0005739 | | 4.27E-13 | | 1672 | | 82 | | 32 | |
| GO:CC | peroxisomal membrane | | GO:0005778 | | 0.00159939 | | 68 | | 82 | | 5 | |
| GO:CC | oxidoreductase complex | | GO:1990204 | | 0.04231137 | | 134 | | 82 | | 5 | |
|  |  |  | |  | |  | |  | |  | |  |
| Pathways depicted by the list of transcripts with inverse association with linc-GALNTL6-4 (g:Profiler) | | | | | | | | | | | | |
| source | term_name | | term_id | | adj_p_value | | term_size | | query_size | | intersec_size | |
| GO:MF | enzyme regulator activity | | GO:0030234 | | 0.010993085 | | 1319 | | 83 | | 17 | |
| GO:BP | anatomical structure morphogenesis | | GO:0009653 | | 0.00059118 | | 2683 | | 80 | | 28 | |
| GO:BP | cell migration | | GO:0016477 | | 0.004166058 | | 1475 | | 80 | | 19 | |
| GO:BP | neuron projection extension | | GO:1990138 | | 0.01221603 | | 181 | | 80 | | 7 | |
| GO:BP | regulation of catalytic activity | | GO:0050790 | | 0.018077497 | | 1788 | | 80 | | 20 | |
| GO:BP | chemotaxis | | GO:0006935 | | 0.020403609 | | 461 | | 80 | | 10 | |
| GO:BP | cell adhesion | | GO:0007155 | | 0.022731164 | | 1504 | | 80 | | 18 | |
| GO:CC | extracellular region | | GO:0005576 | | 3.19E-07 | | 4206 | | 82 | | 40 | |
| GO:CC | collagen-containing extracellular matrix | | GO:0062023 | | 0.000124133 | | 419 | | 82 | | 11 | |
| GO:CC | cell leading edge | | GO:0031252 | | 0.008168333 | | 424 | | 82 | | 9 | |
| GO:CC | membrane | | GO:0016020 | | 0.009736183 | | 9853 | | 82 | | 55 | |
| GO:CC | axon | | GO:0030424 | | 0.038116406 | | 641 | | 82 | | 10 | |
| GO:CC | cell junction | | GO:0030054 | | 0.039169908 | | 2214 | | 82 | | 20 | |
| GO:CC | late endosome | | GO:0005770 | | 0.041448581 | | 305 | | 82 | | 7 | |

**Table S4.** Clinical and biochemical parameters of subjects included in our cross-sectional study.

|  | Non-obese | Obesity | p-value^a^ |
| --- | --- | --- | --- |
| N | 76 | 136 |  |
| Gender (% men) | 26 | 20 |  |
| Age (years) | 49 ± 11 | 46 ± 11 | 0.043 |
| BMI (kg/m^2^) | 25.6 ± 3.7 | 44.1 ± 7.3 | <0.0001 |
| Fat mass (%) | 33.7 ± 7.2 | 55.9 ± 10.3 | <0.0001 |
| SBP (mmHg) | 126.5 ± 16.2 | 136.1 ± 17.9 | <0.0001 |
| DBP (mmHg) | 74.2 ± 11.3 | 79.3 ± 11.2 | 0.002 |
| Fasting glucose (mg/dl) | 94.4 ± 26.5 | 107.3 ± 39.6 | 0.004 |
| HbA1c (%) | 5.5 ± 1.3 | 5.4 ± 1.1 | 0.404 |
| Total cholesterol (mg/dl) | 201.8 ± 39.5 | 192.3 ± 35.6 | 0.049 |
| HDL cholesterol (mg/dl) | 62.2 ± 21.4 | 56.3 ± 30.7 | 0.01 |
| LDL cholesterol (mg/dl) | 119.5 ± 36.8 | 113.2 ± 32.0 | 0.334 |
| Fasting triglycerides (mg/dl) | 103.6 ± 56.3 | 125.8 ± 79.2 | 0.027 |

Data are expressed as mean ± standard deviation (S.D.). Obesity was set at BMI ≥30 kg/m^2^. BMI: body mass index; S/DBP: systolic/diastolic blood pressure; HbA1c: glycated haemoglobin; H/LDL: high/low-density lipoprotein. Two-tailed Student’s *t*-test was used to obtain the p-values.

**Table S5.** Spearmans’ rank (*r*) correlations and multiple regression (β) analysis for linc-GALNTL6-4 in subcutaneous (SC) and omental (OM) adipose tissues versus clinical and biochemical determinations.

|  | SC (n=212) | | | | OM (n=172) | | |
| --- | --- | --- | --- | --- | --- | --- | --- |
| Spearmans’ correlations | r | | p-value | | r | | p-value |
| Age (years) | 0.09 | | 0.193 | | 0.122 | | 0.11 |
| BMI (kg/m^2^) | -0.528 | | <0.0001 | | -0.494 | | <0.0001 |
| Fat mass (%) | -0.458 | | <0.0001 | | -0.187 | | 0.044 |
| SBP (mmHg) | -0.332 | | <0.0001 | | -0.258 | | 0.003 |
| DBP (mmHg) | -0.248 | | 0.001 | | -0.145 | | 0.102 |
| Fasting glucose (mg/dl) | -0.41 | | <0.0001 | | -0.219 | | 0.004 |
| HbA1c (%) | -0.001 | | 0.992 | | 0.072 | | 0.458 |
| Total cholesterol (mg/dl) | 0.009 | | 0.92 | | 0.033 | | 0.68 |
| HDL cholesterol (mg/dl) | 0.333 | | <0.0001 | | 0.164 | | 0.044 |
| LDL cholesterol (mg/dl) | -0.059 | | 0.425 | | 0.079 | | 0.345 |
| Fasting triglycerides (mg/dl) | -0.407 | | <0.0001 | | -0.207 | | 0.009 |
| *LEP* | -0.454 | | <0.0001 | | -0.328 | | 0.002 |
| *TNFα* | -0.487 | | <0.0001 | | -0.368 | | <0.0001 |
| *IRS1* | 0.402 | | <0.0001 | | 0.443 | | <0.0001 |
| *GLUT4* | 0.621 | | <0.0001 | | 0.639 | | <0.0001 |
| *ACACA* | 0.533 | | <0.0001 | | 0.269 | | 0.002 |
| *FASN* | 0.646 | | <0.0001 | | 0.523 | | <0.0001 |
| SC vs. OM | 0.611 | | <0.0001 | |  | |  |
|  |  |  | |  | |  | |
| Multiple linear regression | β | | p-value | | β | | p-value |
| Gender | 0.141 | | 0.025 | | 0.095 | | 0.177 |
| Age (years) | -0.022 | | 0.738 | | 0.001 | | 0.99 |
| BMI (kg/m^2^) | -0.441 | | <0.0001 | | -0.462 | | <0.0001 |
| Fasting glucose (mg/dl) | -0.13 | | 0.048 | | -0.11 | | 0.134 |
| Adjusted R Square | 24.5 (p<0.0001) | | | | 23.5 (p<0.0001) | | |

BMI: body mass index; S/DBP: systolic/diastolic blood pressure; HbA1c: glycated haemoglobin; H/LDL: high/low-density lipoprotein; *LEP*: leptin; *IRS1*: insulin receptor substrate 1; *GLUT4*: glucose transporter type 4; *FASN*: fatty acid synthase; *ACACA*: acetyl-CoA carboxylase alpha; *TNFα*: tumor necrosis factor alpha. Beta (β) is the standardized regression coefficient, which evaluates the relative impact of independent variables in multiple linear regression analyses. Adjusted R square express the percentage of the variance explained by independent variables (i.e., 0.5 is 50%).

**Table S6.** Clinical and biochemical parameters of subjects included in our cross-sectional study from the METSIM cohort (n=335).

|  | Non-obese | Obesity | p_value |
| --- | --- | --- | --- |
| N | 277 | 58 |  |
| Gender | 277 | 58 |  |
| Age (years) | 54.1 ± 4.9 | 54.3 ± 5.1 | 0.836 |
| BMI (kg/m^2^) | 25.6 ± 2.4 | 32.6 ± 3.3 | <0.0001 |
| Fat mass (%) | 20 ± 4.4 | 26.3 ± 4 | <0.0001 |
| SBP (mmHg) | 133 ± 14.9 | 138.2 ± 13.3 | 0.010 |
| DBP (mmHg) | 86.5 ± 8.7 | 91.7 ± 9.3 | 0.0002 |
| Fasting glucose (mg/dl) | 102.7 ± 8.4 | 106.3 ± 10.3 | 0.016 |
| HbA1c (%) | 5.6 ± 0.3 | 5.8 ± 0.4 | 0.0002 |
| Total cholesterol (mg/dl) | 211.8 ± 32.5 | 215.2 ± 36.9 | 0.513 |
| HDL cholesterol (mg/dl) | 57.9 ± 18.8 | 51.1 ± 14 | 0.001 |
| LDL cholesterol (mg/dl) | 134.4 ± 27.7 | 141.1 ± 30.9 | 0.124 |
| Fasting triglycerides (mg/dl) | 117.6 ± 74.1 | 140.2 ± 65.6 | 0.022 |

Data are expressed as mean ± standard deviation (S.D.). Obesity was set at BMI ≥30 kg/m^2^. BMI: body mass index; S/DBP: systolic/diastolic blood pressure; HbA1c: glycated haemoglobin; H/LDL: high/low-density lipoprotein. Two-tailed Student’s *t*-test was used to obtain the p-values.

**Table S7.** Spearmans’ rank (*r*) correlations and multiple regression (β) analysis for linc-GALNTL6-4 in bulk RNA-sequencing of subcutaneous adipose tissue from the METSIM cohort(n=335) versus clinical and biochemical determinations.

|  | SC (n=335) | |
| --- | --- | --- |
| Spearmans’ correlations | r | p-value |
| Age (years) | -0.045 | 0.409 |
| BMI (kg/m^2^) | -0.266 | <0.0001 |
| Fat mass (%) | -0.295 | <0.0001 |
| SBP (mmHg) | -0.131 | 0.017 |
| DBP (mmHg) | -0.065 | 0.239 |
| Fasting glucose (mg/dl) | -0.065 | 0.245 |
| HbA1c (%) | -0.053 | 0.331 |
| Total cholesterol (mg/dl) | -0.032 | 0.563 |
| HDL cholesterol (mg/dl) | 0.123 | 0.024 |
| LDL cholesterol (mg/dl) | -0.054 | 0.323 |
| Fasting triglycerides (mg/dl) | -0.197 | <0.0001 |
| *LEP* | -0.289 | <0.0001 |
| *TNFα* | -0.225 | <0.0001 |
| *IRS1* | 0.319 | <0.0001 |
| *ACACA* | 0.415 | <0.0001 |
| *FASN* | 0.372 | <0.0001 |
|  |  |  |
| Multiple linear regression | β | p-value |
| Age (years) | -0.0095 | 0.386 |
| BMI (kg/m^2^) | -0.0729 | <0.0001 |
| Fasting glucose (mg/dl) | -0.0016 | 0.82 |
| Adjusted R Square | 6.62 (p<0.0001) | |

BMI: body mass index; S/DBP: systolic/diastolic blood pressure; HbA1c: glycated haemoglobin; H/LDL: high/low-density lipoprotein; *LEP*: leptin; *TNFα*: tumor necrosis factor alpha; *IRS1*: insulin receptor substrate 1; *GLUT4*: glucose transporter type 4; *ACACA*: acetyl-CoA carboxylase alpha; *FASN*: fatty acid synthase. Beta (β) is the standardized regression coefficient, which evaluates the relative impact of independent variables in multiple linear regression analyses. Adjusted R square express the percentage of the variance explained by independent variables (i.e., 0.5 is 50%).

**Table S8.** Pathways related to linc-GALNTL6-4 (g:Profiler), according to the gene co-expression network analysis performed in human adipocytes.

| Pathways depicted by the list of transcripts with direct association with linc-GALNTL6-4 | | | | | | | | | | | | |
| --- | --- | --- | --- | --- | --- | --- | --- | --- | --- | --- | --- | --- |
| source | term_name | | term_id | | adj_p_value | | term_size | | query_size | | intersec_size | |
| GO:MF | catalytic activity | | GO:0003824 | | 2.19E-10 | | 5751 | | 493 | | 215 | |
| GO:MF | small molecule binding | | GO:0036094 | | 3.17E-10 | | 2583 | | 493 | | 121 | |
| GO:MF | protein binding | | GO:0005515 | | 0.00011012 | | 14811 | | 493 | | 410 | |
| GO:MF | amide binding | | GO:0033218 | | 0.03240215 | | 376 | | 493 | | 23 | |
| GO:MF | primary active transmembrane transporter | | GO:0015399 | | 0.0434288 | | 170 | | 493 | | 14 | |
| GO:BP | small molecule metabolic process | | GO:0044281 | | 1.61E-18 | | 1846 | | 492 | | 113 | |
| GO:BP | generation of precursor metabolites and energy | | GO:0006091 | | 1.65E-13 | | 514 | | 492 | | 49 | |
| GO:BP | mitochondrion organization | | GO:0007005 | | 4.12E-08 | | 547 | | 492 | | 42 | |
| GO:BP | response to nutrient levels | | GO:0031667 | | 0.01057289 | | 484 | | 492 | | 29 | |
| GO:BP | carnitine metabolic process, CoA-linked | | GO:0019254 | | 0.03191292 | | 3 | | 492 | | 3 | |
| GO:BP | carbohydrate metabolic process | | GO:0005975 | | 0.03829487 | | 573 | | 492 | | 31 | |
| GO:CC | mitochondrion | | GO:0005739 | | 8.36E-39 | | 1672 | | 512 | | 138 | |
|  |  |  | |  | |  | |  | |  | |  |
| Pathways depicted by the list of transcripts with inverse association with linc-GALNTL6-4 | | | | | | | | | | | | |
| source | term_name | | term_id | | adj_p_value | | term_size | | query_size | | intersec_size | |
| GO:MF | protein binding | | GO:0005515 | | 7.51E-18 | | 14811 | | 762 | | 664 | |
| GO:MF | enzyme activator activity | | GO:0008047 | | 1.84E-05 | | 618 | | 762 | | 53 | |
| GO:MF | extracellular matrix structural constituent | | GO:0005201 | | 6.06E-05 | | 167 | | 762 | | 23 | |
| GO:MF | actin binding | | GO:0003779 | | 0.00186244 | | 444 | | 762 | | 38 | |
| GO:MF | death receptor activity | | GO:0005035 | | 0.00276839 | | 13 | | 762 | | 6 | |
| GO:BP | regulation of response to stimulus | | GO:0048583 | | 7.56E-22 | | 3909 | | 756 | | 258 | |
| GO:BP | supramolecular fiber organization | | GO:0097435 | | 6.03E-11 | | 799 | | 756 | | 75 | |
| GO:BP | extracellular matrix organization | | GO:0030198 | | 2.07E-10 | | 320 | | 756 | | 43 | |
| GO:BP | cell junction organization | | GO:0034330 | | 0.00057652 | | 755 | | 756 | | 56 | |
| GO:BP | glomerulus vasculature development | | GO:0072012 | | 0.00078277 | | 28 | | 756 | | 9 | |
| GO:BP | viral entry into host cell | | GO:0046718 | | 0.00189252 | | 156 | | 756 | | 20 | |
| GO:BP | viral process | | GO:0016032 | | 0.00509982 | | 428 | | 756 | | 36 | |
| GO:BP | blood circulation | | GO:0008015 | | 0.00573057 | | 503 | | 756 | | 40 | |
| GO:BP | positive regulation of RNA polymerase II | | GO:0045944 | | 0.00950304 | | 1245 | | 756 | | 76 | |
| GO:BP | negative regulation of RNA polymerase II | | GO:0000122 | | 0.01243215 | | 978 | | 756 | | 63 | |
| GO:BP | antigen processing (...) via MHC class I | | GO:0019885 | | 0.01755005 | | 22 | | 756 | | 7 | |
| GO:BP | antigen processing (...) via MHC class Ib | | GO:0002428 | | 0.02718949 | | 16 | | 756 | | 6 | |
| GO:BP | adaptive immune response based on (...) | | GO:0002460 | | 0.03122359 | | 302 | | 756 | | 27 | |
| GO:BP | DNA damage response by p53 class mediator | | GO:0030330 | | 0.0461197 | | 78 | | 756 | | 12 | |
| GO:CC | cell periphery | | GO:0071944 | | 8.28E-22 | | 6202 | | 784 | | 351 | |
| GO:CC | anchoring junction | | GO:0070161 | | 3.9E-08 | | 903 | | 784 | | 72 | |
| GO:CC | MHC class I protein complex | | GO:0042612 | | 1.69E-05 | | 8 | | 784 | | 6 | |
| GO:CC | actin cytoskeleton | | GO:0015629 | | 0.00022396 | | 501 | | 784 | | 41 | |
| GO:CC | cell leading edge | | GO:0031252 | | 0.00306986 | | 424 | | 784 | | 34 | |
| GO:CC | actin-based cell projection | | GO:0098858 | | 0.01234338 | | 219 | | 784 | | 21 | |
| GO:CC | collagen trimer | | GO:0005581 | | 0.03583643 | | 93 | | 784 | | 12 | |
| GO:CC | main axon | | GO:0044304 | | 0.03890138 | | 67 | | 784 | | 10 | |

**Table S9.** Spearmans’ rank (*r*) correlations and multiple regression (β) analysis for apolipoprotein C1 (*APOC1*) gene expression in SC and omental OM adipose tissues.

|  | APOC1 | | | |
| --- | --- | --- | --- | --- |
|  | SC (n=116) | | OM (n=91) | |
| Spearmans’ correlations | r | p-value | r | p-value |
| Age (years) | -0.011 | 0.905 | 0.172 | 0.042 |
| BMI (kg/m^2^) | -0.032 | 0.738 | 0.142 | 0.097 |
| Fat mass (%) | 0.014 | 0.882 | 0.17 | 0.046 |
| SBP (mmHg) | 0.206 | 0.074 | 0.041 | 0.694 |
| DBP (mmHg) | 0.05 | 0.669 | 0.044 | 0.67 |
| Fasting glucose (mg/dl) | 0.064 | 0.502 | 0.088 | 0.308 |
| HbA1c (%) | 0.207 | 0.081 | 0.181 | 0.09 |
| Total cholesterol (mg/dl) | 0.032 | 0.744 | 0.124 | 0.163 |
| HDL cholesterol (mg/dl) | -0.068 | 0.507 | -0.143 | 0.122 |
| LDL cholesterol (mg/dl) | 0.006 | 0.951 | 0.097 | 0.305 |
| Fasting triglycerides (mg/dl) | 0.297 | 0.002 | 0.207 | 0.02 |
| SC vs. OM | 0.165 | 0.119 |  |  |
|  |  |  |  |  |
| Multiple linear regression | β | p-value | β | p-value |
| Gender | -0.185 | 0.051 | -0.182 | 0.037 |
| Age (years) | 0.283 | 0.003 | 0.185 | 0.036 |
| BMI (kg/m^2^) | 0.161 | 0.085 | 0.133 | 0.135 |
| linc-GALNTL6-4 | 0.24 | 0.011 | 0.257 | 0.003 |
| Adjusted R Square | 17.4 (p=0.001) | | 12.9 (p=0.002) | |

BMI: body mass index; S/DBP: systolic/diastolic blood pressure; HbA1c: glycated haemoglobin; H/LDL: high/low-density lipoprotein. Beta (β) is the standardized regression coefficient, which evaluates the relative impact of independent variables in multiple linear regression analyses. Adjusted R square express the percentage of the variance explained by independent variables (i.e., 0.5 is 50%).

**Table S10.** TaqMan assays used during this research.

| Genes | Ref. #TaqMan assays |
| --- | --- |
| linc-GALNTL6-4 | LNCCLCN343 |
| ADIPOQ | Hs00605917_m1 |
| FABP4 | Hs01086177_m1 |
| FASN | Hs01005622_m1 |
| LEP | Hs00174877_m1 |
| TNFα | Hs00174128_m1 |
| IRS1 | Hs00178563_m1 |
| SLC2A4 | Hs00168966_m1 |
| ACACA | Hs01046047_m1 |
| APOC1 | Hs00155790_m1 |
| 18S rRNA | Hs99999901_s1 |
| RPLP0 | Hs99999902_m1 |
| ACTB | Hs99999903_m1 |
| PPIA | [Hs99999904_m1](https://www.thermofisher.com/taqman-gene-expression/product/Hs99999904_m1?CID=&ICID=&subtype=) |
| GAPDH | Hs99999905_m1 |
